# *SOD-1* Variants in Amyotrophic Lateral Sclerosis: Systematic Re-Evaluation According to ACMG-AMP Guidelines

**DOI:** 10.3390/genes13030537

**Published:** 2022-03-18

**Authors:** Paola Ruffo, Benedetta Perrone, Francesca Luisa Conforti

**Affiliations:** Medical Genetics Laboratory, Department of Pharmacy, Health and Nutritional Sciences, University of Calabria, 87036 Rende, Italy; paolaruffo.bio@gmail.com (P.R.); benedetta.perrone90@gmail.com (B.P.)

**Keywords:** amyotrophic lateral sclerosis, ACMG-AMP guidelines, *SOD1*, re-evaluation, database, pathogenicity

## Abstract

Amyotrophic lateral sclerosis (ALS) is the most common type of motor neuron disease whose causes are unclear. The first ALS gene associated with the autosomal dominant form of the disease was *SOD1*. This gene has a high rate of rare variants, and an appropriate classification is essential for a correct ALS diagnosis. In this study, we re-evaluated the classification of all previously reported *SOD1* variants (*n* = 202) from ALSoD, project MinE, and in-house databases by applying the ACMG-AMP criteria to ALS. New bioinformatics analysis, frequency rating, and a thorough search for functional studies were performed. We also proposed adjusting criteria strength describing how to apply them to *SOD1* variants. Most of the previously reported variants have been reclassified as likely pathogenic and pathogenic based on the modified weight of the PS3 criterion, highlighting how in vivo or in vitro functional studies are determining their interpretation and classification. Furthermore, this study reveals the concordance and discordance of annotations between open databases, indicating the need for expert review to adapt the study of variants to a specific disease. Indeed, in complex diseases, such as ALS, the oligogenic inheritance, the presence of genes that act as risk factors and the reduced penetration must be considered. Overall, the diagnosis of ALS remains clinical, and improving variant classification could support genetic data as diagnostic criteria.

## 1. Introduction

Neurodegenerative diseases are a heterogeneous set of diseases of the central nervous system, characterized by the progressive degeneration of neurons’ structure and function. ALS, also known as Charcot disease or Lou Gehrig’s disease, is the most common motor neuron disease with a global annual incidence ranging from 1 to 3 cases per 100,000 population [1]. It is characterized by the progressive neuromuscular junctions dismantling and the motor neurons degeneration in the motor cortex, brain stem, and spinal cord. Loss of motor neurons leads to progressive paralysis and death from respiratory failure within 3–5 years of the onset of disease [2]. To date, despite ALS being known to be a multifactorial disease associated with genetic predisposition, environmental factors, and lifestyle, its causes are unclear. In 10% of cases, ALS is caused by a genetic mutation that can characterize several generations of the same family (familial ALS (fALS)) or the disease arises suddenly and without any known familial link (sporadic ALS (sALS)) [3]. The main genes directly associated with the onset of the disease and which account for almost 50% of fALS and 6% of sALS cases in the world are *SOD1*, *TARDBP*, *FUS*, and *C9orf72* [4].

The first ALS gene associated with the adult-onset autosomal dominant form of the disease was mapped to the chromosome 21q22.1–22.2 and encodes for the cytoplasmic Cu/Zn superoxide dismutase (*SOD1*). Mutations in this gene were first described and linked to the disease in 1993 [5], and their frequencies range from 1% to 2% and from 13% to 20% in sALS and fALS patients, respectively [6]. *SOD1* gene encodes a homodimer enzyme composed of 154 highly conserved amino acids between species. *SOD1* mutants show a gain of function in which the protein appears to take on a new toxic function, probably related to an increased tendency for mutated proteins to assemble and form aggregates in motor neurons [7].

The advancement of next-generation sequencing (NGS) technologies and the extension of screening tests have made it possible to associate over 100 genes to the disease and thousands of variants [8]. The enormous increase in data and the massive number of unknown variants obtained using these approaches require an interpretative step that allows for clinical classification of the variants as pathogenic or benign. The guidelines proposed in 2015 by the American College of Medical Genetics and Genomics and by the Association for Molecular Pathology (ACMG-AMP) [9] represent the criteria to be used for the classification of variants associated with Mendelian disorder in different categories—namely, benign, likely benign, variants of uncertain significance, likely pathogenic, and pathogenic. Among all, one of the criteria considered very important is based on the characterization of gene variants through functional studies (PS3), and the studies in which experimental tests are carried out are fundamental, as they help the interpretation and classification of pathogenic gene variants.

Since 2015, other publications have highlighted the need to re-evaluate some criteria in the classification of variants [10,11,12]. In particular, detailed guidance was provided to determine the appropriate strength of evidence of functional studies that can be used in the interpretation of clinical variants [13]. However, despite the introduction of these dedicated guidelines and algorithms, the variant interpretation process is complex and not always an efficient process. In addition, the existence of discordant variant classifications across public databases is one of the well-documented limitations in the interpretation of the pathogenicity of variants.

Based on this premise, a periodic re-evaluation of the genetic variants underlying complex diseases is necessary, especially in complex diseases such as ALS where mutation–phenotype correlations are growing rapidly.

To date, over 200 *SOD1* variants have been identified. Most are present in heterozygosity [14], confirming the autosomal dominant inheritance of the gene in this motor neuron disease [15], while others represent an exception, such as the recessive variant D91A (p.Asp91Ala) that shows frequent homozygosity in Scandinavian populations and Nordic countries [16] and the compound heterozygous variant D97N (p.Asp97Asn) [17].

This study provides an updated evaluation of all previously reported *SOD1* variants in the Amyotrophic Lateral Sclerosis Online Genetics Database (ALSoD, https://alsod.ac.uk/, 20 December 2021), project MinE (http://databrowser.projectmine.com/, 22 December 2021) and in-house databases [18] by applying the ACMG-AMP guidelines with criteria adapted for *SOD1*. We also assess the concordance of annotations between open databases (ClinVar and LOVD), highlighting ongoing sources of discordance.

## 2. Materials and Methods

### 2.1. Structured Search

*SOD1* variants associated with ALS were extrapolated from the ALSoD, project MinE, and in-house databases [18]. All these substitutions were from patients diagnosed with definite, probable, and probable lab-supported ALS based on the revised El Escorial criteria [19]. We investigated all the genetic variants listed by ALSoD, while we selected only the variants matching the “LoF + Missense” criterion listed by Project MinE. In addition, a PubMed literature search was conducted using the keywords “*SOD1* variant” and “*SOD1* mutation”. Studies aimed at investigating the functional significance of genetic variants and abstracts related to genetic variants underlying ALS were also considered in the study.

### 2.2. ACMG-AMP Pathogenicity Analysis

The analysis and clinical classification of ALS variants were based on the ACMG-AMP guidelines (Supplementary Table S1). The assigned criteria were then combined, according to the scoring rules expressed in Supplementary Figure S1, to choose a classification from the 5-level system.

### 2.3. Frequency Analysis of Variants

For the evaluation of the minor allelic frequency (MAF), gnomAD v2.1 (gnomad.broadinstitute.org) was used. According to the ACMG-AMP guidelines, if a variant exists in a large general population or a control cohort, it can be considered a criterion 2 moderate pathogen (PM2). Recently, Oza et al. [20] recommended downgrading the weight of the PM2 criterion to a supporting weight in guidance from the Sequence Variant Interpretation (SVI) working group (https://clinicalgenome.org/site/assets/files/5182/pm2_-_svi_recommendation_-_approved_sept2020.pdf, 27 January 2022). In our context, *SOD1* variants completely absent from all databases or with a MAF < 0.001 met the moderate pathogen criterion 2 (PM2) [21,22].

### 2.4. Computational Tools Analysis

The pathogenicity of the variants was also evaluated using multiple in silico genomic tools, including SIFT [23], Polyphen-2 [24], MutationTaster [25], MutationAssessor [26], FATHMM [27], PROVEAN [28], and CADD [29]. Splicing prediction analysis was performed using SpiP and SpliceAI tools [30]. The convergence should be considered as supporting evidence of pathogenicity or benignity. An adverse consequence predicted with more than six tools is considered to be evidence to support pathogenicity (PP3), while at least three total and concordant predictors are required to support a benign state (BP4). However, if the in silico predictions disagree, this test should not be used for the classification of a variant.

### 2.5. Analysis of Functional Studies

Functional studies conducted in vitro or in vivo may show a detrimental effect on protein function, supporting the pathogenicity of the tested variant (PS3), or no effect, thus supporting a benign impact of the variant (BS3). As recommended by Brnich et al., several factors must be considered for the assignment of this criterion [13]. It is necessary to delineate the genetic mechanism of the disease to choose the appropriate functional analysis, as well as the experimental model to be used on which to perform functional tests. Assays performed must be coded in biological replicates, and an adequate number of control cases should be considered. Finally, the test result must be in line with the pathogenetic mechanism of the disease [13].

ALS is a complex biological system, and the precise mechanisms underlying selective cell death in the disease are unknown. Current understanding indicates that there may be a complex interplay between genetic factors, oxidative stress, excitotoxicity, protein aggregation, impaired axonal transport, and mitochondrial damage. Over the years, several in vitro and in vivo experimental models have been exploited to study the molecular processes underlying motor neuron degradation and disease progression, providing essential small pieces to the ALS puzzle [31,32]. However, only an integrated and complimentary use of different experimental models could improve our understanding of the pathogenic mechanism of the disease.

According to Brnich’s recommendations and experimental evidence on *SOD1* variants, we assigned three different weights to the PS3 criterion: PS3_Strong, PS3_Moderate, and PS3_Supporting. PS3_Strong was assigned to variants in which in vivo functional assay results showed toxic *SOD1* function and disruption of mitochondrial dynamics, protein folding, axonal transport, and cellular metabolism. The PS3_Moderate and PS3_Supporting criteria were assigned to variants investigated by functional in vitro studies, following the flowchart shown in Supplementary Figure S2.

### 2.6. Critical Functional Domain Position

**Figure 1:**
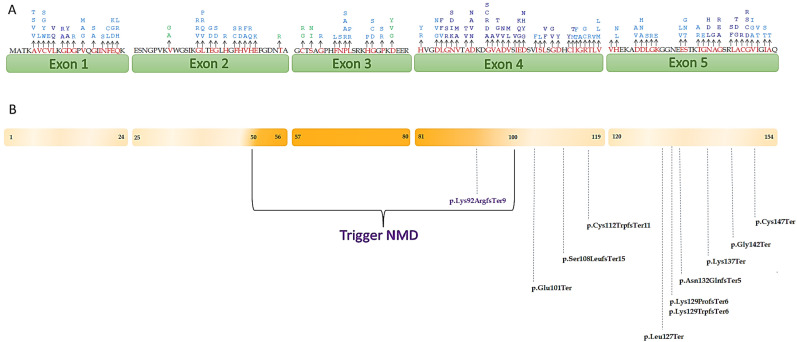
(**A**) Schematic representation of *SOD1* showing the localization of variants that were assigned PM1. The amino acid changes are represented in red. The different weights of the criterion are represented by the different colors: dark blue, PM1_Strong; light blue, PM1_Moderate; green, PM1_Supporting. The numbers under the exons represent the amino acid distribution; (**B**) localization of variants that undergo/do not undergo NMD. The variants localized in *SOD1* region where PTCs trigger NMD are represented in purple, the variants localized in the region that do not undergo NMD are colored in black.

Pathogenic *SOD1* variants associated with ALS are spread over the entire length of the gene. Hotspot mutations are characterized by the presence of many missense variants, in-frame, and nonsynonymous variants, most of which have a pathogenicity classification. The universal protein source, UniProt (https://www.uniprot.org/, 29 January 2022), and bioinformatics tools can help identify mutations in established hotspots or functional domains. The PM1 rule considers reported variants within the domain and is assigned with the ratio pathogenic to total nonpathogenic variants is greater than 1.5, with at least 5 pathogenic variants within the domain. We established that the PM1 criterion can be upgraded to strong (PM1_Strong) when at least 14 pathogenic variants are reported in the functional domain; the moderate weight (PM1_Moderate) of this criterion was assigned in the presence of at least 7 pathogenic missense variants. Finally, we considered PM1_Supporting when 5–6 pathogenic mutations are reported in the domain (Supplementary Table S2). The distribution of *SOD1* variants is shown in Figure 1A.

### 2.7. Evaluation of the PVS1 Criterion

A strong weight in the classification of ALS genes is represented by the PVS1 criterion. This is attributed to a “Null variant in a gene where loss of function (LOF) is a known mechanism of disease”. Gain of function and several mechanisms are confirmed to be involved in ALS [33,34,35]. However, much evidence about *SOD1* loss of function (LOF) suggests it may play a role in ALS pathogenesis, probably increasing the susceptibility to neurodegeneration. Overall, the major effect of *SOD1* mutations in ALS is linked to the protein aggregation but these may also lead to loss of function of *SOD1* activity or loss of the nuclear function, where *SOD1* acts as a transcription factor [6].

According to the recommendations provided by the ClinGen SVI working group [10] and taking into account Guissart’s considerations [36], we adapted ACMG-AMP guidelines for *SOD1* by evaluating the right strength related to the PVS1 criterion and distinguishing the variants with PVS1_Very strong and PVS1_Strong. In particular, we assigned PVS1_Very strong criterion when variants do not undergo the nonsense-mediated mRNA decay (NMD) and PVS1_Strong when variants undergo NMD. A representation of the *SOD1* gene showing the localization of premature termination codons (PTCs) that cause ALS and NMD escaping is reported in Figure 1B.

### 2.8. Comparison between Databases

To assess the concordance between annotations generated by ClinVar and LOVD databases, we compared a list of 202 variants of the *SOD1* gene across several online resources. The databases investigated generate an automated interpretation of the variants. Specifically, Leiden Open Variation Database (LOVD—https://www.lovd.nl/, 5 January 2022) is a software database for collecting and visualizing variants in the DNA sequence [37]. ClinVar (https://www.ncbi.nlm.nih.gov/clinvar/, 7 January 2022) is an archive that aggregates information on genomic variation and the various correlations with human health [38].

### 2.9. Statistical Analysis

The chi-square concordance test was performed for discordance–concordance and variant classification across the various databases, using the SPSS 20.0 test (SPSS, Chicago, IL, USA). Pathogenic/likely pathogenic *SOD1* variants were compared using Fisher’s test. A *p*-value < 0.05 was considered statistically significant.

## 3. Results

### 3.1. Summary of Variants

In this study, 202 sequence variants of *SOD1* (NM_000454.5) were collected and analyzed. These variants have a MAF < 0.001 considering gnomAD 2.1 populations. Table 1 lists all variant sequences investigated and corresponding classification according to the ACMG-AMP guidelines.

**Figure 2:**
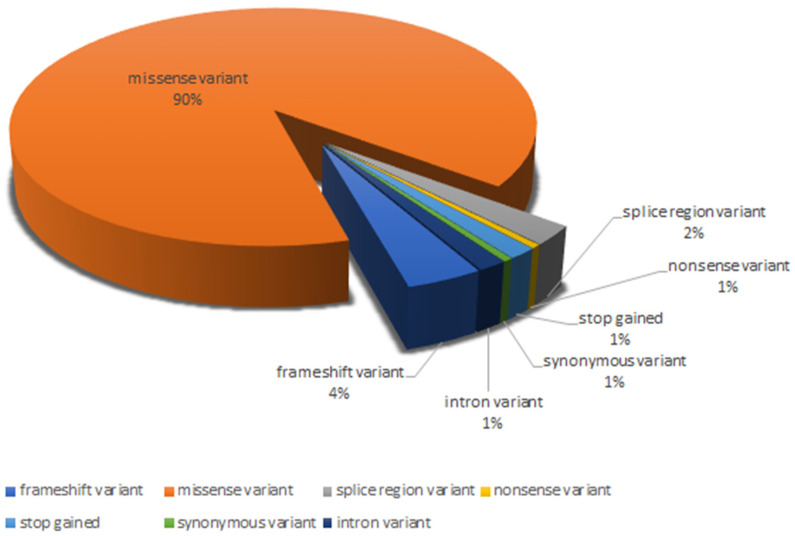
Distribution of the different variant types analyzed.

The variants analyzed were five splice regions (2%), one nonsense (1%), eight frameshifts (4%), three stop-gained (1%), one synonymous (1%), and three intron variants (1%), while all remaining were missense variants (90%) (Figure 2).

In total, 13 variants (6.4%) were classified as variants of uncertain significance (VUS), with insufficient or conflicting evidence regarding the role of molecular alteration in disease. Of these, nine were missense variants (c.59A>G, c.89T>C, c.179G>A, c.298A>G, c.172T>C, c.179G>T, c.328G>T, c.35A>C, c.172T>G), three were intron variants (c.358-11A>G, c.358-304C>G, c.358-10T>G) and one was a splice region variant (c.240-7T>G). The variants classified as likely pathogenic were 136 (67.3%), of which 2 were frameshifts (1.5%), 1 was stop-gained (0.7%), 3 were splice region variants (2.2%), and 130 were missense variants (95.6%). The remaining 53 (26.3%) were considered pathogenic and included 6 frameshifts (11.3%), 1 splice region (1.9%), 2 nonsense (3.8%), 2 stop-gained (3.8%), 1 synonymous variant (1.9%), and 41 missense variants (77.3%).

### 3.2. Contribution of Each Criterion to the Final Classification

**Figure 3:**
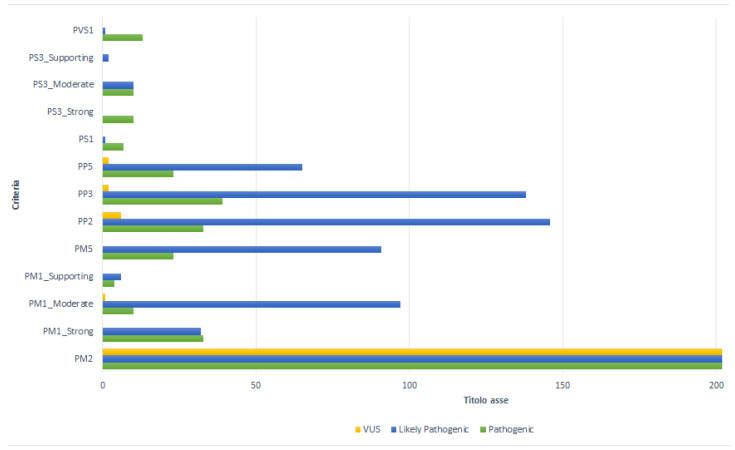
Contribution of each criterion to the final classification.

The contribution of each criterion to the final classification is shown in Figure 3. The PM2 criterion was assigned to all 202 variants analyzed in this study. Pathogenic variants of the *SOD1* gene are often identified in a single patient or an isolated family. This evidence underlines the rarity of the variants and therefore allows the PM2 criterion to acquire a moderate level of evidence of pathogenicity.

The PM1 criterion was assigned to 186 variants (92%), with different weights depending on the number of pathogenic variants located in the functional domain of the gene. The PM1_Strong rule was assigned to 68 variants (36.6%), of which 32 (47.1%) were classified as likely pathogenic, 33 (48.5%) as pathogenic, and 3 (4.4%) as VUS. The PM1_ Moderate was assigned to 108 variants (58%); in this category, 97 (89.9%) were likely pathogenic, 10 (9.3%) as pathogenic, and only 1 (0.9%) was classified as VUS. The PM1_Supporting weight was assigned to 10 mutations (5.4%), of which 6 terms (60%) were classified as likely pathogenic and 4 (40%) as VUS.

The PP2 criterion characterized 185 variants (91.6%): 146 (79%) as likely pathogenic, 33 (17.9%) as pathogenic, and 6 (3.1%) as VUS.

Computational tools predicted a disease-causing role (PP3) in 179 (88.6%) cases examined. Of these variants, 138 variants (77%) were classified as likely pathogenic, 39 (21.8%) as pathogenic, and 2 (1.1) as VUS.

In the present study, the PM5 criterion assigned to a “novel missense change at an aminoacidic residue where a different missense change determined to be pathogenic has been seen before” [9], showed a moderate impact in 114 variants (56.4%). Of these, 91 (79.8%) were likely pathogenic, while the remaining 23 (20%) were pathogenic.

According to the ACMG-AMP guidelines, the PP5 criterion was assigned to 90 variants (90/202; 44.6%) for which different studies were available in the literature. The presence of the PP5 criterion allowed the classification of 65 (72.2%) likely pathogenic, 23 (25.5%) pathogenic, and of the remaining 2 (2.2%) VUS variants. The splice region c.240-7T>G and the intron c.358-11A>G variants were characterized as VUS, with the PM2 and PP5 criteria, since no data from the literature were available.

Considering previously published functional studies for the evaluation of the variant effect and the impact of the mutation on the clinical phenotype, the PS3 criterion was assigned to 32 (32/202; 15.8%) variants (Supplementary Table S3). PS3_Strong was assigned to 10 (31.3%) variants classified as pathogenic. These pathogenic variants were characterized by the attendance of multiple functional studies performed in various experimental models (Supplementary Table S3) [39,40,41,42,43,44,45,46,47,48,49]. The p.Gly94Ala mutation, known as G93A, was used to create the first ALS-associated *SOD1* transgenic mouse. This model, which expresses large amounts of mutant *SOD1*, develops adult-onset neurodegeneration of spinal motor neurons and progressive motor deficits leading to paralysis, and it is now widely used both for pathogenic molecular mechanisms and for preclinical studies useful for the specific therapy of affected patients [31,39,40,41]. This mutation is currently the one studied in the largest number of animal models including C. Elegans, Saccharomyces Cerevisiae, Swine, and Danio rerio (Zebrafish) models. In the same way, experimental models of the transgenic mouse G93A and H46R (p.His47arg) show degeneration of the upper and lower motoneurons associated with a gravity directly proportional to the altered protein expression [31,39,42]. In addition, in vivo experiments show that *SOD1*-D91A (p.Asp91Ala), the most common mutation affecting *SOD1* linked to ALS, is less toxic than other tested mutants, while homozygous mice develop fatal motor neuron disease similar to that observed in homozygous-D91A human ALS patients [41,43].

A Zebrafish model with G94R (p.Gly94Arg) variant has been developed, and this shows a mutated expression of *SOD1* at a physiological level, which allows targeted pre-clinical studies [31,49]. For the variants G86R (p.Gly86Arg) [31,45,46,47] and G38R (p.Gly38Arg) [31,47,48], there are several functional studies carried out on transgenic mouse models in which there are high expression levels of the mutated gene and differences in the age of onset and progression of the disease as well as lifespan. For the G86R variant, studies have also been carried out using the C. Elegans nematode model. In particular, the presence of this missense variant produces an abnormal function of neurons ad a cellular dysfunction at the muscular level. The same variant was tested on Drosophila melanogaster models showing a progressive deterioration of motor functions with a specific clinical phenotype. The same experimental model was also used to study the effect of variants G38R and I114T (p.Ile114thr), determining a decrease in the lifespan of the midge accompanied by abnormalities in movement (Supplementary Table S3).

The effect of other mutations, classified with the PS3_Moderate or PS3_Supporting criterion, was evaluated by functional in vitro studies [50,51,52,53,54,55,56,57,58,59,60,61,62,63] assessing the correlation between the specific genetic alteration and the modification of oxidative stress, mitochondrial and cellular metabolism in different clinical phenotypes (Supplementary Table S3). PS3 with moderate weight was attributed to 20 (62.5%) variants: 10 (50%) were classified as likely pathogenic, while the remaining 10 (50%) as pathogenic. Finally, the PS3_Supporting weight was assigned to two (6.2%) variants classified as likely pathogenic (Supplementary Figure S2).

Another criterion with a strong impact on the classification, the PS1, was assigned to only eight *SOD1* variants (4%). Of these, seven (87.5%) were classified as pathogenic and one (12.5%), the c.355G>T, was classified as likely pathogenic.

In our study, it was possible to attribute the PVS1 rule to 11 (5.4%) variants, 10 classified as pathogenic (92.8%) and 1 as likely pathogenic (7.1%). Only the variant c.275_276delAA expected to undergo NMD, while the other 10 mutations (c.301G>T, c.441T>A, c.409A>T, c.380T>A, c.424G>T, c.383_384insACCC, c.384_385insTGGG, c.320_321insT, c.335dup, c.383_392dup) did not undergo NMD, presenting the PVS1_Very strong criterion. The stop-gain variant c.301G>T, although known as a pathogenic mechanism (PVS1) and absent from population databases (PM2), was classified as likely pathogenic (Supplementary Table S4).

The frameshift variants c.272_274dupACA and c.56_58delTCA cause a change in the length of the protein for which the PM4 criterion can be applied. Combined with the PM and PP criteria, these two variants were classified as likely pathogenic.

### 3.3. Concordance–Discordance between Open Databases and Reclassification Based on ACMG-AMP Guidelines

LOVD contains a total of 47 variants, of which only 18 are classified as pathogenic, likely pathogenic, or VUS. The comparison of these variants (18/202, 9%) revealed a discordant classification (*p*-value < 0.001) in two substitutions. The variant c.59A>G, defined as likely benign in LOVD databases, was reclassified as VUS, while the c.272A>C was reclassified from VUS to pathogenic (Supplementary Table S5).

Evaluating *SOD1* variant classifications in ClinVar database, 72 (35.6%) were concordant, and 19 (9.4%) variants were discordant (*p*-value < 0.001). In particular, 12 VUS were reclassified as likely pathogenic (*n* = 10) and pathogenic (*n* = 3), while 1 pathogenic variant was reclassified as VUS. Seven variants, defined as conflicting interpretations of pathogenicity by ClinVar, were reclassified: six as likely pathogenic/pathogenic and only one as VUS (Supplementary Table S5). Finally, 111 terms (55%), searched for rs ID, did not match in ClinVar.

## 4. Discussion

In this study, for the first time, ALS-associated *SOD1* variants were systematically re-evaluated to perform clinical interpretation according to the ACMG-AMP guidelines. We also provided an updated assessment of variant concordance–discordance between bioinformatics tools used to evaluate variants’ roles and the criteria application provided by the above-mentioned guidelines adapted for *SOD1*.

Previous research has reported that only 34% of variants have a concordant classification according to the ACMG-AMP guidelines [64]. The high percentage of discordance between classifications can be explained by how individual laboratories interpret and analyze variants data, based on published levels of evidence and the misuse of ACMG-AMP guidelines.

Various scientific evidence supports the idea that ALS is caused by a combination of genetic and environmental factors [65,66]. To understand and quantify the value of the contribution of genetics in the pathogenesis of the disease several studies have been conducted [46]. Although a large number of genes have been related to ALS, other variants are to be discovered, which could play adjunct roles in increasing risk or susceptibility to the disease. Specific mutations not identifiable by GWAS or massive sequencing methods, such as repeated expansions [67] or variants in noncoding regions, could belong to this category [68,69].

Human *SOD1* is widely known to be an important protein in the antioxidant system. The main hallmark of fALS is the presence of *SOD1* aggregates in the spinal cord of patients [70], and the chemically modified form of the protein in the spinal cord of sporadic ALS patients has also been found [71]. *SOD1* mutations were the first to be directly associated with the disease pathogenesis and the classification of variants in such a complex pathology remains a challenge. The heterogeneity of clinical data and the difference in approaches used for the interpretation of variants are the main challenges in variant classification [72].

**Figure 4:**
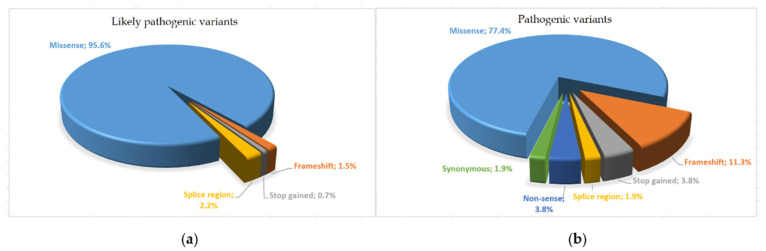
Pie chart of different variant distribution types identified: (**a**) likely pathogenic variants; (**b**) pathogenic variants.

*SOD1* gene has a high rate of rare variants, and an appropriate classification is essential for a correct ALS diagnosis. Furthermore, one of the greatest challenges in interpreting pre-existing assessments is the heterogeneity in the approaches used for variant analysis. In this study, we applied a standardized in silico analysis and assessed the frequency of the variants, as well as considering the presence of functional and segregation studies. Results showed that 136 (67.3%) variants met the criteria of likely pathogenic, 53 (26.3%) were classified as pathogenic (Figure 4), and the remaining variants (*n* = 13; 6.4%) were categorized as VUS.

In our study, the PS3 criterion proved to be fundamental for the pathogenicity classification of 32 variants, representing 62.5% of total pathogenic terms. However, the near absence of functional and segregation data for a large number of terms remains an important limiting factor, considering that functional evidence is the main scoring driver for pathogenic/likely pathogenic for missense variants. Compared with all the variants analyzed, it is clear that there are few functional studies, conducted in vitro or in vivo, which show a detrimental effect on the protein. Furthermore, the PS3 criterion is not automatically assigned in the various databases, as functional evidence must be searched by the geneticist consulting PubMed and other available sites (Mastermind, etc.). After the manual revision, the strength of the criterion can also be changed according to the relevance of the evidence.

The attribution of the PM1 criterion was based on scientific evidence showing the absence of specific mutational hotspot mutations in *SOD1* linked to ALS, indicating the whole protein involved [73]. In *SOD1*, several variants have been described to cluster in all five exons of the gene identified as hotspots (Figure 1A). Rather than defining clear function domains, these regions are defined by an increase in pathogenic variations identified in ALS patients. For this reason, we considered three different levels of strength of the criterion: PM1_Strong (≥14 variants), PM1_Moderate (at least 7 variants), and PM1_Supporting (5–6 variants).

All of the variants present in this study with a MAF value less than 0.001 and absents from all databases were considered to have a moderate pathogenicity level. The definition of a MAF threshold for ALS, in which significant confounding factors coexist (late-onset of the disease, age-dependent, variant-related reduced penetrance, and oligogenic inheritance), cannot ignore many factors difficult to evaluate, such as the inheritance model (monoallelic for most *SOD1*-ALS variants), the prevalence of the disease, and the penetrance. Failing consensus on allele frequency thresholds, the PM2 criterion was assigned to these cases.

For most of the missense variants involved in our study and classified as pathogenic or likely pathogenic, the PP5 criterion was considered supporting evidence with a very strong value, as the variants are present in several studies in the literature. However, this criterion must be used with caution as “the evidence is not available to the laboratory to perform an independent evaluation“ [11].

A strong impact on the evaluation of a mutation is expressed by the PS1 criterion, which is attributed when the same amino acid variation of a well-known causative variant occurs. The variant could act directly through the specific change of DNA or could modify the protein sequence through various mechanisms, such as an alteration in splicing. To support the pathogenicity of genetic variants, it is necessary to have well-established and reproducible functional studies.

The PVS1 criterion is considered the greatest impact, characterized by identifying a variant that causes a loss-of-function (LoF) allele. Although the gain of function is causative for amyotrophic lateral sclerosis, *SOD1* loss of function may play a role in ALS, contributing to the onset and progression of the disease [73,74]. Many nonsense or frameshift mutations may lead to a LOF of *SOD1* by affecting its structures and/or interactions pattern [6]. mRNA containing premature termination codons are known to be rapidly degraded by nonsense-mediated mRNA decay and premature termination codons that escape NMD in *SOD1*, causing the production of truncated *SOD1* protein, which may have high toxicity [36] (Figure 1B).

An element of increasing importance is constituted by databases that report identified variants with their associated phenotypes and the interpretation of their functional role. In ALS, this concept is even more important since the vast majority of the variants identified are new and/or private, and for this reason, it is very important to share the knowledge obtained in dedicated databases. Another aspect to note is the presence/absence of *SOD1* variants in these different databases. This study shows that many variants of this gene are absent in online archives used and, therefore, stresses that it is necessary to revise these data, so they are also open source, allowing the scientific community to search for updated information of genetic mutations, which are extremely important to the disease. A comparison of the annotations between open databases and our reclassification based on ACMG-AMP guidelines with the criteria adapted for *SOD1* revealed a different rate of discordance. In particular, comparing ClinVar, we found discordance between pathogenic/likely pathogenic variants and VUS in 9.4% of terms. A case in point is the most common *SOD1* mutation, p.Asp91Ala, which is still reported in ClinVar with conflicting interpretations of pathogenicity despite extensive evidence demonstrating pathogenicity according to the ACMG-AMP guidelines. In this public repository, many variants are associated with classification according to the ACMG-AMP guidelines. This archive accepts data derived from different working groups, the relevant literature, and documents from other web resources. To ensure the quality of the declarations, a review phase is carried out that involves evaluation by groups of experts. The variant data are then updated based on the new information presented, and therefore, an adequate time is required for the process. [39].

Based on the data obtained, it is evident that the mere application of the guidelines for interpreting genetic variants developed and refined so far cannot be considered sufficient in ALS to address various problems related to the implementation of new genetic analysis technologies in a diagnostic and clinical context. A manual reinterpretation of variants is essential to adapt the study to a particular disease, considering the specific characteristics of the gene and allowing for a consistent application. In this way, the different ACMG-AMP criteria acquire a different weight based on the type of pathology, scientific evidence, and knowledge of the variant itself.

## 5. Conclusions

In this study, we re-evaluated the classification of all previously reported *SOD1* variants (*n* = 202) in ALSoD, Project MinE, and in-house databases by applying the ACMG-AMP criteria to ALS. Our results underline the importance of classifying variants involved in ALS and *SOD1* variants with caution.

Specific guidelines for ALS would allow a more adequate classification of the large number of variants of uncertain significance, which have been constantly discovered. This can be accomplished by carrying out genetic research on large cohorts of patients and controls and by sharing the data obtained with the scientific community. Furthermore, it is crucial to develop and implement new analysis strategies that could facilitate the discovery and the validation of genetic factors involved in the pathogenesis of this disease that is still unknown. To date, the diagnosis of ALS remains, based on clinical, not genetic criteria. The improvement of the existing variant classification systems available could provide a better basis for using genetic findings as diagnostic criteria.

## Figures and Tables

**Table 1:** *SOD1* variants in Amyotrophic Lateral Sclerosis: Systematic re-evaluation according to ACMG-AMP guidelines.

Coding Change (NM_000454.5)	Protein Change (NP_000445.1)	Variant ID	Type	PS4	PM3	PP4	PM1	PM2	PP2	PM4	PM5	PM6	PP1	PP3	PP5	PS1	PS2	PS3	PVS1	Benign Criteria	Re-Classification
c.13G>A	p.Ala5Thr	rs121912444	Missense variant				*M	X	X		X			X	X			*S			P
c.13G>T	p.Ala5Ser	rs121912444	Missense variant				*M	X	X		X			X	X						P
c.13_14delGCinsTT	p.Ala5Phe		Missense variant				*M	X	X		X			X							LP
c.14C>T	p.Ala5Val	rs121912442	Missense variant				*M	X	X		X			X	X			*S			P
c.16G>T	p.Val6Leu	rs1568807314	Missense variant				*M	X	X		X			X							LP
c.19T>A	p.Cys7Ser	rs1312702973	Missense variant				*M	X	X		X			X							LP
c.19T>G	p.Cys7Gly	rs1312702973	Missense variant				*M	X	X		X			X							LP
c.20G>A	p.Cys7Tyr	rs121912448	Missense variant				*M	X	X		X			X							LP
c.21C>G	p.Cys7Trp		Missense variant				*M	X	X		X			X							LP
c.23T>A	p.Val8Glu	rs1568807330	Missense variant				*M	X	X		X				X						LP
c.25C>G	p.Leu9Val	rs1568807333	Missense variant				*S	X	X		X			X	X						P
c.26T>A	p.Leu9Gln	rs1568807342	Missense variant				*S	X	X		X			X	X						P
c.31G>C	p.Gly11Arg	rs1568807350	Missense variant				*S	X	X					X							LP
c.32G>C	p.Gly11Ala	rs1555836167	Missense variant				*S	X	X					X							LP
c.34G>T	p.Asp12Tyr	rs762628133	Missense variant				*S	X	X					X							LP
c.35A>C	p.Asp12Ala	rs1568807374	Missense variant				*S	X	X												VUS
c.37G>C	p.Gly13Arg	rs121912456	Missense variant				*M	X	X					X	X						LP
c.43G>A	p.Val15*Met	rs1568807400	Missense variant				*M	X	X		X			X	X			*M			LP
c.44T>C	p.Val15Ala	rs1202989817	Missense variant				*M	X	X		X										LP
c.44T>G	p.Val15Gly	rs1202989817	Missense variant				*M	X	X		X			X				*M			LP
c.49G>A	p.Gly17Ser	rs121912453	Missense variant				*M	X	X		X			X	X						LP
c.50G>C	p.Gly17Ala	rs1200906022	Missense variant				*M	X	X		X			X	X						LP
c.358-304C>G		rs1555836889	intron variant					X													VUS
c.56_58delTCA	p.Ile19del		frameshift variant				*M	X		X				X							LP
c.59A>G	p.Asn20Ser	rs768029813	Missense variant				*M	X	X												VUS
c.62T>G	p.Phe21Cys	rs1555836169	Missense variant				*M	X	X					X	X						LP
c.63C>G	p.Phe21Leu	rs1555836170	Missense variant				*M	X	X		X			X							LP
c.64G>A	p.Glu22Lys	rs121912450	Missense variant				*M	X	X		X				X			*M			LP
c.65A>G	p.Glu22Gly	rs1568807435	Missense variant				*M	X	X		X			X				*M			LP
c.66G>C	p.Glu22Asp		Missense variant				*M	X	X		X			X							LP
c.68A>T	p.Gln23Leu	rs1169198442	Missense variant				*M	X	X		X			X							LP
c.68A>G	p.Gln23Arg	rs1169198442	Missense variant				*M	X	X		X			X							LP
c.69G>C	p.Gln23His	rs1424217272	Missense variant				*M	X	X		X			X							LP
c.89T>C	p.Val30Ala	rs1568809118	Missense variant					X	X					X							VUS
c.95T>C	p.Val32Ala	rs1428716759	Missense variant				*H	X	X					X	X						LP
c.95T>G	p.Val32Gly	rs1428716759	Missense variant				*H	X	X		X			X							LP
c.112G>A	p.Gly38Arg	rs121912431	Missense variant				*M	X	X					X	X	X		*S			P
c.112G>C	p.Gly38Arg	rs121912431	Missense variant				*M	X	X					X	X	X		*S			P
c.113G>T	p.Gly38Val	rs1555836517	Missense variant				*M	X	X		X			X							LP
c.115C>G	p.Leu39Val	rs121912432	Missense variant				*M	X	X		X			X	X						LP
c.116T>A	p.Leu39Gln	rs1555836520	Missense variant				*M	X	X		X			X							LP
c.116T>G	p.Leu39Arg	rs1555836520	Missense variant				*M	X	X		X			X				*H			LP
c.116T>C	p.Leu39Pro		Missense variant				*M	X	X		X			X							LP
c.122A>G	p.Glu41Gly	rs1568809149	Missense variant				*M	X	X					X							LP
c.123A>T	p.Glu41Asp		Missense variant				*M	X	X					X							LP
c.124G>A	p.Gly42Ser	rs121912433	Missense variant				*M	X	X		X			X	X			*M			LP
c.125G>A	p.Gly42Asp	rs121912434	Missense variant				*M	X	X		X			X	X			*M			LP
c.131A>G	p.His44Arg	rs121912435	Missense variant				*M	X	X					X	X						LP
c.137T>C	p.Phe46Ser	rs121912457	Missense variant				*M	X	X		X			X							LP
c.137T>G	p.Phe46Cys	rs121912457	Missense variant				*M	X	X					X	X						LP
c.139C>G	p.His47Asp	rs748897491	Missense variant				*M	X	X		X			X							LP
c.140A>G	p.His47Arg	rs121912443	Missense variant				*M	X	X					X	X			*S			P
c.142G>T	p.Val48Phe	rs1555836523	Missense variant				*M	X	X					X							LP
c.143T>C	p.Val48Ala	rs1568809169	Missense variant				*M	X	X					X							LP
c.146A>G	p.His49Arg	rs1568809172	Missense variant				*M	X	X		X			X	X						LP
c.147T>G	p.His49Gln	rs1568809175	Missense variant				*M	X	X		X			X	X						LP
c.148G>A	p.Glu50Lys	rs1568809178	Missense variant				*M	X	X					X							LP
c.164C>G	p.Thr55Arg	rs986277034	Missense variant				*H	X	X					X	X						LP
c.172T>C	p.Cys58Arg	rs1568810255	Missense variant				*H	X	X					X							VUS
c.172T>G	p.Cys58Gly		Missense variant				*H	X	X					X							VUS
c.179G>A	p.Ser60Asn	rs1413388444	Missense variant				*H	X	X					X							VUS
c.179G>T	p.Ser60Ile	rs1413388444	Missense variant				*H	X	X					X							VUS
c.184G>C	p.Gly62Arg	rs1568810268	Missense variant				*M	X	X					X							LP
c.193T>C	p.Phe65Leu	rs1030039318	Missense variant				*M	X	X					X	X						LP
c.197A>G	p.Asn66Ser	rs1568810275	Missense variant				*M	X	X		X			X							LP
c.199C>G	p.Pro67Ala	rs1356474292	Missense variant				*M	X	X		X			X							LP
c.199C>T	p.Pro67Ser	rs1356474292	Missense variant				*M	X	X		X			X							LP
c.199G>C	p.Pro67Ala	rs1356474292	Missense variant				*M	X	X		X			X							LP
c.200C>G	p.Pro67Arg	rs1568810284	Missense variant				*M	X	X		X			X							LP
c.203T>C	p.Leu68Pro	rs1568810289	Missense variant				*M	X	X		X				X					BP4	LP
c.203T>G	p.Leu68Arg	rs1568810289	Missense variant				*M	X	X		X										LP
c.215A>C	p.His72Pro		Missense variant				*M	X	X		X			X							LP
c.217G>A	p.Gly73Ser	rs121912455	Missense variant				*M	X	X					X	X						LP
c.217G>T	p.Gly73Cys	rs121912455	Missense variant				*M	X	X		X			X							LP
c.218G>A	p.Gly73Asp		Missense variant				*M	X	X		X			X							LP
c.223C>T	p.Pro75Ser		Missense variant				*M	X	X					X				*H			LP
c.224C>G	p.Pro75Arg		Missense variant				*M	X	X					X							LP
c.229G>T	p.Asp77Tyr	rs1601157750	Missense variant				*H	X	X		X			X	X						LP
c.230A>T	p.Asp77Val	rs1568810316	Missense variant				*H	X	X		X			X	X						LP
c.230A>G	p.Asp77Gly		Missense variant				*H	X	X		X			X							LP
c.240-7T>G		rs1568810602	splice region					X							X						VUS
c.241C>T	p.His81Tyr		Missense variant				*M	X	X		X			X							LP
c.242A>G	p.His81Arg	rs121912458	Missense variant				*M	X	X		X			X	X						LP
c.250G>A	p.Asp84Asn	rs1555836789	Missense variant				*M	X	X					X							LP
c.251A>T	p.Asp84Val		Missense variant				*M	X	X					X							LP
c.251A>G	p.Asp84Gly	rs1568810615	Missense variant				*M	X	X					X							LP
c.253T>G	p.Leu85Val	rs121912452	Missense variant				*M	X	X		X			X	X			*S		BP7	P
c.255G>C	p.Leu85Phe	rs1315541036	Missense variant				*S	X	X		X			X	X	X					P
c.255G>T	p.Leu85Phe		Missense variant				*S	X	X		X			X		X					P
c.256G>A	p.Gly86Ser	rs121912436	synonymous variant				*S	X	X		X			X	X						P
c.256G>C	p.Gly86Arg	rs121912436	Missense variant				*S	X	X		X			X	X			*S			P
c.259A>G	p.Asn87Asp	rs1555836792	Missense variant				*S	X	X					X	X						LP
c.260A>G	p.Asn87Ser	rs11556620	Missense variant				*S	X	X					X	X			*M			P
c.260A>T	p.Asn87Ile	rs11556620	Missense variant				*M	X	X					X	X						LP
c.261T>A	p.Asn87Lys	rs1555836793	Missense variant				*S	X	X		X			X							P
c.262G>A	p.Val88Met	rs1568810641	Missense variant				*S	X	X		X			X	X						P
c.263T>C	p.Val88Ala	rs1339283341	Missense variant				*S	X	X		X			X	X						P
c.268G>A	p.Ala90Thr	rs1568810660	Missense variant				*S	X	X		X			X	X						P
c.269C>T	p.Ala90Val	rs1280042397	Missense variant				*S	X	X		X			X	X						P
c.271G>A	p.Asp91Asn	rs1343616996	Missense variant				*S	X	X		X									BP4	LP
c.272A>C	p.Asp91Ala	rs80265967	Missense variant				*S	X	X		X				X			*S			P
c.272A>T	p.Asp91Val	rs80265967	Missense variant				*S	X	X		X										P
c.272_274dupACA	p.Asp91_Lys92 insAsn		frameshift variant				*S	X		X											LP
c.275_276delAA	p.Lys92ArgfsTer9		frameshift variant					X											*S		P
c.280G>A	p.Gly94Ser	rs121912437	Missense variant				*S	X	X		X			X				*M			P
c.280G>T	p.Gly94Cys	rs121912437	Missense variant				*S	X	X		X			X	X						P
c.280G>C	p.Gly94Arg	rs121912437	Missense variant				*S	X	X		X			X	X			*S			P
c.281G>A	p.Gly94Asp	rs121912438	Missense variant				*S	X	X		X			X	X			*M			P
c.281G>C	p.Gly94Ala	rs121912438	Missense variant				*S	X	X		X			X	X			*S			P
c.284T>C	p.Val95Ala	rs202198235	Missense variant				*S	X	X					X							LP
c.286G>A	p.Ala96Thr	rs1568810686	Missense variant				*S	X	X		X			X							LP
c.287C>G	p.Ala96Gly	rs1568810690	Missense variant				*S	X	X					X	X						LP
c.287C>T	p.Ala96Val	rs1568810690	Missense variant				*S	X	X					X	X						LP
c.289G>A	p.Asp97Asn	rs121912459	Missense variant				*S	X	X		X									BP4	LP
c.290A>T	p.Asp97Val	rs1555836803	Missense variant				*S	X	X						X					BP4	LP
c.292G>A	p.Val98*Met	rs1555836806	Missense variant				*S	X	X					X	X						LP
c.292G>C	p.Val98Leu	rs1555836806	Missense variant				*S	X	X		X			X							LP
c.298A>G	p.Ile100Val	rs760740095	Missense variant				*S	X	X												VUS
c.301G>A	p.Glu101Lys	rs76731700	Missense variant				*S	X	X		X				X			*M			P
c.301G>T	p.Glu101Ter	rs76731700	stop gained					X											*VS		P
c.301G>C	p.Glu101Gln		Missense variant				*S	X	X		X										LP
c.302A>G	p.Glu101Gly	rs121912439	Missense variant				*S	X	X		X			X	X			*M			P
c.304G>A	p.Asp102Asn	rs1568810715	Missense variant				*S	X	X		X			X	X						P
c.304G>C	p.Asp102His	rs1568810715	Missense variant				*S	X	X		X			X	X						P
c.304G>T	p.Asp102Ty	rs1568810715	Missense variant				*S	X	X		X			X							LP
c.305A>G	p.Asp102Gly	rs1568810721	Missense variant				*S	X	X		X			X	X			*M			P
c.313A>T	p.Ile105Phe	rs121912445	Missense variant				*M	X	X					X	X						LP
c.317C>T	p.Ser106Leu	rs1378590183	Missense variant				*M	X	X					X	X						LP
c.319C>G	p.Leu107Val	rs121912440	Missense variant				*S	X	X					X	X						LP
c.319C>T	p.Leu107Phe	rs121912440	Missense variant				*S	X	X		X			X							LP
c.320_321insT	p.Ser108LeufsTer15		frameshift variant					X						X					*VS		P
c.326G>A	p.Gly109Glu	rs1359299834	Missense variant				*S	X	X		X			X							LP
c.326G>T	p.Gly109Val	rs1359299834	Missense variant				*S	X	X					X	X						P
c.328G>T	p.Asp110Tyr	rs567432143	Missense variant				*S	X	X												VUS
c.335G>A	p.Cys112Tyr	rs1601158483	Missense variant				*S	X	X					X	X						LP
c.335dup	p.Cys112TrpfsTer11	rs1568810771	frameshift variant					X						X	X				*VS		P
c.338T>C	p.Ile113Thr	rs74315452	Missense variant				*S	X	X		X			X	X						P
c.339C>G	p.Ile113*Met	rs1299542356	Missense variant				*M	X	X		X			X							LP
c.340A>T	p.Ile114Phe	rs1568810780	Missense variant				*M	X	X		X			X							LP
c.341T>C	p.Ile114Thr	rs121912441	Missense variant				*M	X	X					X	X			*M			LP
c.344G>C	p.Gly115Ala	rs1568810789	Missense variant				*M	X	X		X			X							LP
c.346C>G	p.Arg116Gly	rs1301635320	Missense variant				*M	X	X		X			X	X						LP
c.346C>T	p.Arg116Cys	rs1301635320	Missense variant				*M	X	X		X			X							LP
c.358-11A>G		rs369600566	intron variant					X							X						VUS
c.358-10T>G		rs1197141604	intron variant					X						X							VUS
c.350C>G	p.Thr117Arg	rs1568810800	Missense variant				*M	X	X					X							LP
c.352C>G	p.Leu118Val	rs199474723	Missense variant				*M	X	X									*M			LP
c.355G>C	p.Val119Leu	rs1235629842	Missense variant				*M	X	X					X	X	X					P
c.355G>T	p.Val119Leu	rs1235629842	Missense variant				*M	X	X					X	X	X					LP
c.355G>A	p.Val119Met		Missense variant				*M	X	X					X	X						LP
c.358G>T	p.Val120Phe		Missense variant				*M	X	X					X							LP
c.358G>C	p.Val120Leu	rs1457889952	Missense variant				*M	X	X		X			X							LP
c.361C>A	p.His121Asn		Missense variant				*M	X	X					X							LP
c.362A>T	p.His121Leu	rs1410925719	Missense variant				*M	X	X					X							LP
c.374A>C	p.Asp125Ala	rs1568811366	Missense variant				*M	X	X		X			X							LP
c.374A>T	p.Asp125Val	rs1568811366	Missense variant				*M	X	X		X			X	X			*M			LP
c.376G>C	p.Asp126His	rs1568811372	Missense variant				*M	X	X		X			X	X						LP
c.376G>A	p.Asp126Asn		Missense variant				*M	X	X		X			X	X						LP
c.377A>C		rs1164911383	Missense variant				*M	X	X		X			X	X						LP
c.380T>A	p.Leu127Ter	rs121912454	stop gained					X						X	X				*VS		P
c.380T>C	p.Leu127Ser	rs121912454	Missense variant				*M	X	X					X	X						LP
c.382G>C	p.Gly128Arg	rs1568811389	Missense variant				*M	X	X					X							LP
c.383_384insACCC	p.Lys129ProfsTer6		frameshift variant					X						X					*VS		P
c.383_392dup	p.Asn132GlnfsTer5		frameshift variant					X						X	X				*VS		P
c.384_385insTGGG	p.Lys129TrpfsTer6		frameshift variant					X						X					*VS		P
c.385A>G	p.Lys129Glu		Missense variant				*M	X	X					X							LP
c.400G>A	p.Glu134Lys		Missense variant				*M	X	X					X	X						LP
c.401A>T	p.Glu134Val	rs1568811426	Missense variant				*M	X	X		X			X							LP
c.403A>G	p.Ser135Gly	rs1555836932	Missense variant				*M	X	X		X			X							LP
c.404G>A	p.Ser135Asn	rs121912451	Missense variant				*M	X	X					X	X						LP
c.404G>C	p.Ser135Thr	rs121912451	Missense variant				*M	X	X		X			X							LP
c.409A>T	p.Lys137Ter	rs1555836934	nonsense variant					X						X					*VS		P
c.412A>G	p.Thr138Ala	rs1568811445	Missense variant				*M	X	X		X			X							LP
c.413C>G	p.Thr138Arg	rs1568811454	Missense variant				*M	X	X		X			X							LP
c.416G>A	p.Gly139Glu	rs1568811464	Missense variant				*M	X	X					X							LP
c.418A>C	p.Asn140His	rs1568811471	Missense variant				*S	X	X		X			X							LP
c.418A>G	p.Asn140Asp	rs1568811471	Missense variant				*S	X	X		X			X							LP
c.420C>A	p.Asn140Lys	rs1804449	Missense variant				*S	X	X		X			X	X	X		*M			P
c.422C>G	p.Ala141Gly	rs1555836937	Missense variant				*S	X	X					X							LP
c.424G>T	p.Gly142Ter	rs1319062081	stop gained					X						X					*VS		P
c.424G>A	p.Gly142Arg		Missense variant				*S	X	X					X							LP
c.425G>A	p.Gly142Glu	rs1568811489	Missense variant				*S	X	X					X							LP
c.425G>C	p.Gly142Ala	rs1568811489	Missense variant				*S	X	X					X							LP
c.434T>C	p.Leu145Ser	rs121912446	Missense variant				*S	X	X		X			X	X			*M			P
c.435G>C	p.Leu145Phe	rs1482760341	Missense variant				*S	X	X		X			X	X	X		*M			P
c.436G>A	p.Ala146Thr	rs121912447	Missense variant				*S	X	X		X			X	X						P
c.437C>A	p.Ala146Asp	rs1131690781	Missense variant				*S	X	X		X			X	X						P
c.437C>G	p.Ala146Gly	rs1131690781	Missense variant				*S	X	X		X			X							LP
c.439T>C	p.Cys147Arg	rs1568811515	Missense variant				*S	X	X					X				*M			P
c.441T>A	p.Cys147Ter		nonsense variant					X						X					*VS		P
c.442G>C	p.Gly148Arg	rs1568811520	Missense variant				*S	X	X		X			X	X						P
c.442G>A	p.Gly148Ser		Missense variant				*S	X	X		X			X							LP
c.442G>T	p.Gly148Cys		Missense variant				*S	X	X		X			X							LP
c.443G>A	p.Gly148Asp	rs1555836950	Missense variant				*S	X	X		X			X	X						P
c.445G>A	p.Val149Ile	rs567511139	Missense variant				*M	X	X		X			X	X						LP
c.446T>G	p.Val149Gly	rs1476760624	Missense variant				*M	X	X		X			X	X			*M			LP
c.446T>C	p.Val149Ala		Missense variant				*M	X	X		X			X							LP
c.448A>G	p.Ile150Val	rs1169917994	Missense variant				*M	X	X		X			X							LP
c.449T>C	p.Ile150Thr	rs1424014997	Missense variant				*M	X	X					X	X						LP
c.455T>C	p.Ile152Thr	rs121912449	Missense variant				*M	X	X					X	X						LP
c.455T>G	p.Ile152Ser		Missense variant				*M	X	X					X	X						LP
c.457G>A	p.Ala153Thr	rs747094021	Missense variant				*M	X	X					X							LP

*M = *Moderate, *H = Supporting, *S = Strong, *VS = Very strong. LP = Likely pathogenic, P = Pathogenic, VUS = Variant of uncertain significance.

## Data Availability

Not applicable.

## Supplementary Materials

The following supporting information can be downloaded at: https://www.mdpi.com/article/10.3390/genes13030537/s1. Table S1: The ACMG-AMP criteria and their relevance in ALS, Table S2: Assessment of the PM1 criterion in *SOD1* variants, Table S3: List of in vivo and in vitro functional studies considered for the PS3 criterion attribution, Table S4: Characteristics of the *SOD1* variants relevant to the PVS1 criterion, Table S5: List of *SOD1* variants and concordance–discordance between open databases and reclassification, Figure S1: ACMG-AMP guidelines for combining criteria to classify sequencing variants, Figure S2: Decision tree for functional evidence PS3.
