# Supplementary material for: SOD-1 Variants in Amyotrophic Lateral Sclerosis: Systematic Re-Evaluation According to ACMG-AMP Guidelines
Source: Genes (Basel). 2022 Mar 18;13(3):537. doi: 10.3390/genes13030537 (PMC8955492; doi:10.3390/genes13030537)
Supplement: Supplementary file 1 [file genes-13-00537-s001.zip › Supplementary Table S1.pdf]

Supplementary Table S1. The ACMG-AMP criteria and their relevance in ALS.

Acronym	Ranks of pathogenicity impact evidence	Criteria	Relevance in ALS*
**PVS1**	Very strong	Null variant (nonsense, frameshift, canonical +/−1 or 2 splice sites, initiation codon, single or multi-exon deletion) in a gene where loss of function (LOF) is a known mechanism of disease	↑↑
**PS1**	Strong	Same amino acid change as a previously established pathogenic variant regardless of nucleotide change	↑↑
**PS2**	Strong	De novo (both maternity and paternity confirmed) in a patient with the disease and no family history	↑
**PS3**	Strong	Well-established in vitro or in vivo functional studies supportive of a damaging effect on the gene or gene product	↑
**PS4**	Strong	The prevalence of the variant in affected individuals is significantly increased compared to the prevalence in controls	↑↑
**PM1**	Moderate	Located in a mutational hot spot and/or critical and well-established functional domain (e.g. active site of an enzyme) without benign variation	↑↑
**PM2**	Moderate	Absent from controls (or at extremely low frequency if recessive) in Exome Sequencing Project, 1000 Genomes or ExAC Caveat: Population data for indels may be poorly called by next sequencing generation	↑
**PM3**	Moderate	For recessive disorders, detected in trans with a pathogenic variant	↑
**PM4**	Moderate	Protein length changes due to in-frame deletions/insertions in a non-repeat region or stop-loss variants	↑
**PM5**	Moderate	Novel missense change at an amino acid residue where a different missense change determined to be pathogenic has been seen before	↑↑
**PM6**	Moderate	Assumed de novo, but without confirmation of paternity and maternity	↑
**PP1**	Supporting	Co-segregation with disease in multiple affected family members in a gene definitively known to cause the disease	↑↑
**PP2**	Supporting	Missense variant in a gene that has a low rate of benign missense variation and where missense variants are a common mechanism of disease	↑↑
**PP3**	Supporting	Multiple lines of computational evidence support a deleterious effect on the gene or gene product (conservation, evolutionary, splicing impact, etc)	↑↑
**PP4**	Supporting	Patient's phenotype or family history is highly specific for a disease with a single genetic etiology	↑
**PP5**	Supporting	Reputable source recently reports variant as pathogenic but the evidence is not available to the laboratory to perform an independent evaluation	↑↑
**Acronym**	**Ranks of benign impact evidence**	**Criteria**	**Relevance in ALS**
**BA1**	Stand-Alone	Allele frequency is above 5% in Exome Sequencing Project, 1000 Genomes,or ExAC	↑↑
**BS1**	Strong	Allele frequency is greater than expected for disorder	↑↑
**BS2**	Strong	Observed in a healthy adult individual for a recessive (homozygous), dominant (heterozygous), or X-linked (hemizygous) disorder with full penetrance expected at an early age	X
**BS3**	Strong	Well-established in vitro or in vivo functional studies shows no damaging effect on protein function or splicing	↑
**BS4**	Strong	Lack of segregation in affected members of a family	↑/X
**BP1**	Supporting	Missense variant in a gene for which primarily truncating variants are known to cause disease	X
**BP2**	Supporting	Observed in trans with a pathogenic variant for a fully penetrant dominant gene/disorder; or observed in cis with a pathogenic variant in any inheritance pattern	↑/X

**BP3**	Supporting	In-frame deletions/insertions in a repetitive region without a known function	X
**BP4**	Supporting	Multiple lines of computational evidence suggest no impact on gene or gene product (conservation, evolutionary, splicing impact, etc)	↑↑
**BP5**	Supporting	Variant found in a case with an alternate molecular basis for disease	↑
**BP6**	Supporting	Reputable source recently reports variant as benign but the evidence is not available to the laboratory to perform an independent evaluation	↑↑
**BP7**	Supporting	A synonymous (silent) variant for which splicing prediction algorithms predict no impact to the splice consensus sequence nor the creation of a new splice site AND the nucleotide is not highly conserved	↑↑

* The relevance in ALS was previously described [7]. ↑↑: criteria with a more relevant impact in ALS patients. ↑: less related criteria for the classification of the ALS variants. ↑/X: criteria for which precautions should be considered
