# Supplementary material for: SOD-1 Variants in Amyotrophic Lateral Sclerosis: Systematic Re-Evaluation According to ACMG-AMP Guidelines
Source: Genes (Basel). 2022 Mar 18;13(3):537. doi: 10.3390/genes13030537 (PMC8955492; doi:10.3390/genes13030537)
Supplement: Supplementary file 1 [file genes-13-00537-s001.zip › Supplementary Table S2.pdf]

Supplementary Table S2. Assessment of the PM1 criterion in *SOD1* variants.

Coding change (NM_000454.5)	Protein change (NP_000445.1)	Variant ID	PM1_Strong	PM1_Moderate	PM1_Supporting
**c.13G>A**	p.Ala5Thr	rs121912444		13 missense/in-frame/non-synonymous variants (11 pathogenic, 2 uncertain, and 0 benign)	
**c.13G>T**	p.Ala5Ser	rs121912444		13 missense/in-frame/non-synonymous variants (11 pathogenic, 2 uncertain, and 0 benign)	
**c.13_14delGCinsTT**	p.Ala5Phe			13 missense/in-frame/non-synonymous variants (11 pathogenic, 2 uncertain, and 0 benign)	
**c.14C>T**	p.Ala5Val	rs121912442		13 missense/in-frame/non-synonymous variants (11 pathogenic, 2 uncertain, and 0 benign)	
**c.16G>T**	p.Val6Leu	rs1568807314		13 missense/in-frame/non-synonymous variants (11 pathogenic, 2 uncertain, and 0 benign)	
**c.19T>A**	p.Cys7Ser	rs1312702973		15 missense/in-frame/non-synonymous variants (13 pathogenic, 2 uncertain, and 0 benign)	
**c.19T>G**	p.Cys7Gly	rs1312702973		15 missense/in-frame/non-synonymous variants (13 pathogenic, 2 uncertain, and 0 benign)	
**c.20G>A**	p.Cys7Tyr	rs121912448		15 missense/in-frame/non-synonymous variants (13 pathogenic, 2 uncertain, and 0 benign)	
**c.21C>G**	p.Cys7Trp			15 missense/in-frame/non-synonymous variants (13 pathogenic, 2 uncertain, and 0 benign)	
**c.23T>A**	p.Val8Glu	rs1568807330		15 missense/in-frame/non-synonymous variants (13 pathogenic, 2 uncertain, and 0 benign)	
**c.25C>G**	p.Leu9Val	rs1568807333	17 missense/in-frame/non-synonymous variants (15 pathogenic, 2 uncertain, and 0 benign)		
**c.26T>A**	p.Leu9Gln	rs1568807342	17 missense/in-frame/non-synonymous variants (15 pathogenic, 2 uncertain, and 0 benign)		
**c.31G>C**	p.Gly11Arg	rs1568807350	18 missense/in-frame/non-synonymous variants (15 pathogenic, 3 uncertain, and 0 benign)		

**c.32G>C**	p.Gly11Ala	rs1555836167	18 missense/in-frame/non-synonymous variants (15 pathogenic, 3 uncertain, and 0 benign)		
**c.34G>T**	p.Asp12Tyr	rs762628133	19 missense/in-frame/non-synonymous variants (15 pathogenic, 4 uncertain, and 0 benign)		
**c.35A>C**	p.Asp12Ala	rs1568807374	19 missense/in-frame/non-synonymous variants (15 pathogenic, 4 uncertain, and 0 benign)		
**c.37G>C**	p.Gly13Arg	rs121912456		19 missense/in-frame/non-synonymous variants (15 pathogenic, 4 uncertain, and 0 benign)	
**c.43G>A**	p.Val15Met	rs1568807400		19 missense/in-frame/non-synonymous variants (14 pathogenic, 5 uncertain, and 0 benign)	
**c.44T>C**	p.Val15Ala	rs1202989817		19 missense/in-frame/non-synonymous variants (14 pathogenic, 5 uncertain, and 0 benign)	
**c.44T>G**	p.Val15Gly	rs1202989817		19 missense/in-frame/non-synonymous variants (14 pathogenic, 5 uncertain, and 0 benign)	
**c.49G>A**	p.Gly17Ser	rs121912453		17 missense/in-frame/non-synonymous variants (12 pathogenic, 5 uncertain, and 0 benign)	
**c.50G>C**	p.Gly17Ala	rs1200906022		17 missense/in-frame/non-synonymous variants (12 pathogenic, 5 uncertain, and 0 benign)	
**c.56_58delTCA**	p.Ile19del			16 missense/in-frame/non-synonymous variants (11 pathogenic, 5 uncertain, and 0 benign)	
**c.59A>G**	p.Asn20Ser	rs768029813		15 missense/in-frame/non-synonymous variants (10 pathogenic, 5 uncertain, and 0 benign),	
**c.62T>G**	p.Phe21Cys	rs1555836169		14 missense/in-frame/non-synonymous variants (9 pathogenic, 5 uncertain, and 0 benign)	
**c.63C>G**	p.Phe21Leu	rs1555836170		12 missense/in-frame/non-synonymous variants (8 pathogenic, 4 uncertain, and 0 benign)	
**c.64G>A**	p.Glu22Lys	rs121912450		12 missense/in-frame/non-synonymous variants (8 pathogenic, 4 uncertain, and 0 benign)	

**c.65A>G**	p.Glu22Gly	rs1568807435		11 missense/in-frame/non-synonymous variants (8 pathogenic, 3 uncertain, and 0 benign)	
**c.66G>C**	p.Glu22Asp			11 missense/in-frame/non-synonymous variants (8 pathogenic, 3 uncertain, and 0 benign)	
**c.68A>T**	p.Gln23Leu	rs1169198442		11 missense/in-frame/non-synonymous variants (8 pathogenic, 3 uncertain, and 0 benign)	
**c.68A>G**	p.Gln23Arg	rs1169198442		11 missense/in-frame/non-synonymous variants (8 pathogenic, 3 uncertain, and 0 benign)	
**c.69G>C**	p.Gln23His	rs1424217272		10 missense/in-frame/non-synonymous variants (7 pathogenic, 3 uncertain, and 0 benign)	
**c.95T>C**	p.Val32Ala	rs1428716759			8 missense/in-frame/non-synonymous variants (5 pathogenic, 3 uncertain, and 0 benign)
**c.95T>G**	p.Val32Gly	rs1428716759			8 missense/in-frame/non-synonymous variants (5 pathogenic, 3 uncertain, and 0 benign)
**c.112G>A**	p.Gly38Arg	rs121912431		13 missense/in-frame/non-synonymous variants (9 pathogenic, 4 uncertain, and 0 benign)	
**c.112G>C**	p.Gly38Arg	rs121912431		13 missense/in-frame/non-synonymous variants (9 pathogenic, 4 uncertain, and 0 benign)	
**c.113G>T**	p.Gly38Val	rs1555836517		12 missense/in-frame/non-synonymous variants (9 pathogenic, 3 uncertain, and 0 benign)	
**c.115C>G**	p.Leu39Val	rs121912432		13 missense/in-frame/non-synonymous variants (10 pathogenic, 3 uncertain, and 0 benign)	
**c.116T>A**	p.Leu39Gln	rs1555836520		13 missense/in-frame/non-synonymous variants (10 pathogenic, 3 uncertain, and 0 benign)	
**c.116T>G**	p.Leu39Arg	rs1555836520		13 missense/in-frame/non-synonymous variants (10 pathogenic, 3 uncertain, and 0 benign)	
**c.116T>C**	p.Leu39Pro			13 missense/in-frame/non-synonymous variants (10 pathogenic, 3 uncertain, and 0 benign)	

**c.122A>G**	p.Glu41Gly	rs1568809149		15 missense/in-frame/non-synonymous variants (11 pathogenic, 4 uncertain, and 0 benign)	
**c.123A>T**	p.Glu41Asp			15 missense/in-frame/non-synonymous variants (12 pathogenic, 3 uncertain, and 0 benign)	
**c.124G>A**	p.Gly42Ser	rs121912433		15 missense/in-frame/non-synonymous variants (12 pathogenic, 3 uncertain, and 0 benign)	
**c.125G>A**	p.Gly42Asp	rs121912434		15 missense/in-frame/non-synonymous variants (12 pathogenic, 3 uncertain, and 0 benign)	
**c.131A>G**	p.His44Arg	rs121912435		15 missense/in-frame/non-synonymous variants (12 pathogenic, 3 uncertain, and 0 benign)	
**c.137T>C**	p.Phe46Ser	rs121912457		14 missense/in-frame/non-synonymous variants (12 pathogenic, 2 uncertain, and 0 benign)	
**c.137T>G**	p.Phe46Cys	rs121912457		14 missense/in-frame/non-synonymous variants (12 pathogenic, 2 uncertain, and 0 benign)	
**c.139C>G**	p.His47Asp	rs748897491		13 missense/in-frame/non-synonymous variants (11 pathogenic, 2 uncertain, and 0 benign)	
**c.140A>G**	p.His47Arg	rs121912443		13 missense/in-frame/non-synonymous variants (11 pathogenic, 2 uncertain, and 0 benign),	
**c.142G>T**	p.Val48Phe	rs1555836523		11 missense/in-frame/non-synonymous variants (9 pathogenic, 2 uncertain, and 0 benign)	
**c.143T>C**	p.Val48Ala	rs1568809169		11 missense/in-frame/non-synonymous variants (9 pathogenic, 2 uncertain, and 0 benign)	
**c.146A>G**	p.His49Arg	rs1568809172		11 missense/in-frame/non-synonymous variants (9 pathogenic, 2 uncertain, and 0 benign)	
**c.147T>G**	p.His49Gln	rs1568809175		11 missense/in-frame/non-synonymous variants (9 pathogenic, 2 uncertain, and 0 benign)	
**c.148G>A**	p.Glu50Lys	rs1568809178		10 missense/in-frame/non-synonymous variants (9 pathogenic, 1 uncertain, and 0 benign)	

**c.164C>G**	p.Thr55Arg	rs986277034			6 missense/in-frame/non-synonymous variants (5 pathogenic, 1 uncertain, and 0 benign)
**c.172T>C**	p.Cys58Arg	rs1568810255			6 missense/in-frame/non-synonymous variants (5 pathogenic, 1 uncertain, and 0 benign)
**c.172T>G**	p.Cys58Gly				6 missense/in-frame/non-synonymous variants (5 pathogenic, 1 uncertain, and 0 benign)
**c.179G>A**	p.Ser60Asn	rs1413388444			6 missense/in-frame/non-synonymous variants (6 pathogenic, 0 uncertain, and 0 benign)
**c.179G>T**	p.Ser60Ile	rs1413388444			6 missense/in-frame/non-synonymous variants (6 pathogenic, 0 uncertain, and 0 benign)
**c.184G>C**	p.Gly62Arg	rs1568810268		7 missense/in-frame/non-synonymous variants (7 pathogenic, 0 uncertain, and 0 benign)	
**c.193T>C**	p.Phe65Leu	rs1030039318		10 missense/in-frame/non-synonymous variants (9 pathogenic, 1 uncertain, and 0 benign)	
**c.197A>G**	p.Asn66Ser	rs1568810275		11 missense/in-frame/non-synonymous variants (9 pathogenic, 2 uncertain, and 0 benign)	
**c.199C>G**	p.Pro67Ala	rs1356474292		11 missense/in-frame/non-synonymous variants (9 pathogenic, 2 uncertain, and 0 benign)	
**c.199C>T**	p.Pro67Ser	rs1356474292		11 missense/in-frame/non-synonymous variants (9 pathogenic, 2 uncertain, and 0 benign)	
**c.199G>C**	p.Pro67Ala	rs1356474292		17 amino-acids has 11 missense/in-frame/non-synonymous variants (9 pathogenic, 2 uncertain, and 0 benign)	
**c.200C>G**	p.Pro67Arg	rs1568810284		11 missense/in-frame/non-synonymous variants (9 pathogenic, 2 uncertain, and 0 benign),	
**c.203T>C**	p.Leu68Pro	rs1568810289		11 missense/in-frame/non-synonymous variants (9 pathogenic, 2 uncertain, and 0 benign)	
**c.203T>G**	p.Leu68Arg	rs1568810289		11 missense/in-frame/non-synonymous variants (9 pathogenic, 2 uncertain, and 0 benign)	

**c.215A>C**	p.His72Pro			13 missense/in-frame/non-synonymous variants (11 pathogenic, 2 uncertain, and 0 benign)	
**c.217G>A**	p.Gly73Ser	rs121912455		13 missense/in-frame/non-synonymous variants (11 pathogenic, 2 uncertain, and 0 benign)	
**c.217G>T**	p.Gly73Cys	rs121912455		13 missense/in-frame/non-synonymous variants (11 pathogenic, 2 uncertain, and 0 benign)	
**c.218G>A**	p.Gly73Asp			13 missense/in-frame/non-synonymous variants (11 pathogenic, 2 uncertain, and 0 benign)	
**c.223C>T**	p.Pro75Ser			10 missense/in-frame/non-synonymous variants (8 pathogenic, 2 uncertain, and 0 benign)	
**c.224C>G**	p.Pro75Arg			10 missense/in-frame/non-synonymous variants (8 pathogenic, 2 uncertain, and 0 benign)	
**c.229G>T**	p.Asp77Tyr	rs1601157750			7 missense/in-frame/non-synonymous variants (5 pathogenic, 2 uncertain, and 0 benign)
**c.230A>T**	p.Asp77Val	rs1568810316			7 missense/in-frame/non-synonymous variants (5 pathogenic, 2 uncertain, and 0 benign)
**c.230A>G**	p.Asp77Gly				7 missense/in-frame/non-synonymous variants (5 pathogenic, 2 uncertain, and 0 benign)
**c.241C>T**	p.His81Tyr			10 missense/in-frame/non-synonymous variants (10 pathogenic, 0 uncertain, and 0 benign)	
**c.242A>G**	p.His81Arg	rs121912458		10 missense/in-frame/non-synonymous variants (10 pathogenic, 0 uncertain, and 0 benign)	
**c.250G>A**	p.Asp84Asn	rs1555836789		15 missense/in-frame/non-synonymous variants (14 pathogenic, 1 uncertain, and 0 benign)	
**c.251A>T**	p.Asp84Val		15 missense/in-frame/non-synonymous variants (14 pathogenic, 1 uncertain, and 0 benign)		
**c.251A>G**	p.Asp84Gly	rs1568810615		15 missense/in-frame/non-synonymous variants (14 pathogenic, 1 uncertain, and 0 benign)	

**c.253T>G**	p.Leu85Val	rs121912452		15 missense/in-frame/non-synonymous variants (14 pathogenic, 1 uncertain, and 0 benign)	
**c.255G>C**	p.Leu85Phe	rs1315541036	17 missense/in-frame/non-synonymous variants (16 pathogenic, 1 uncertain, and 0 benign)		
**c.255G>T**	p.Leu85Phe		17 missense/in-frame/non-synonymous variants (16 pathogenic, 1 uncertain, and 0 benign)		
**c.256G>A**	p.Gly86Ser	rs121912436	20 missense/in-frame/non-synonymous variants (19 pathogenic, 1 uncertain, and 0 benign)		
**c.256G>C**	p.Gly86Arg	rs121912436	20 missense/in-frame/non-synonymous variants (19 pathogenic, 1 uncertain, and 0 benign)		
**c.259A>G**	p.Asn87Asp	rs1555836792	20 missense/in-frame/non-synonymous variants (19 pathogenic, 1 uncertain, and 0 benign)		
**c.260A>G**	p.Asn87Ser	rs11556620	20 missense/in-frame/non-synonymous variants (19 pathogenic, 1 uncertain, and 0 benign)		
**c.260A>T**	p.Asn87Ile	rs11556620	20 missense/in-frame/non-synonymous variants (19 pathogenic, 1 uncertain, and 0 benign)		
**c.261T>A**	p.Asn87Lys	rs1555836793	20 missense/in-frame/non-synonymous variants (19 pathogenic, 1 uncertain, and 0 benign)		
**c.262G>A**	p.Val88Met	rs1568810641	21 missense/in-frame/non-synonymous variants (20 pathogenic, 1 uncertain, and 0 benign)		
**c.263T>C**	p.Val88Ala	rs1339283341	21 missense/in-frame/non-synonymous variants (20 pathogenic, 1 uncertain, and 0 benign)		
**c.268G>A**	p.Ala90Thr	rs1568810660	22 missense/in-frame/non-synonymous variants (20 pathogenic, 2 uncertain, and 0 benign)		
**c.269C>T**	p.Ala90Val	rs1280042397	22 missense/in-frame/non-synonymous variants (20 pathogenic, 2 uncertain, and 0 benign)		
**c.271G>A**	p.Asp91Asn	rs1343616996	22 missense/in-frame/non-synonymous variants (20 pathogenic, 2 uncertain, and 0 benign)		

**c.272A>C**	p.Asp91Ala	rs80265967	22 missense/in-frame/non-synonymous variants (20 pathogenic, 2 uncertain, and 0 benign)		
**c.272A>T**	p.Asp91Val	rs80265967	22 missense/in-frame/non-synonymous variants (20 pathogenic, 2 uncertain, and 0 benign)		
**c.272_274dupACA**	p.Asp91_Lys92insAsn		23 missense/in-frame/non-synonymous variants (21 pathogenic, 2 uncertain, and 0 benign)		
**c.280G>A**	p.Gly94Ser	rs121912437	26 missense/in-frame/non-synonymous variants (24 pathogenic, 2 uncertain, and 0 benign)		
**c.280G>T**	p.Gly94Cys	rs121912437	26 missense/in-frame/non-synonymous variants (24 pathogenic, 2 uncertain, and 0 benign)		
**c.280G>C**	p.Gly94Arg	rs121912437	26 missense/in-frame/non-synonymous variants (24 pathogenic, 2 uncertain, and 0 benign)		
**c.281G>A**	p.Gly94Asp	rs121912438	24 missense/in-frame/non-synonymous variants (22 pathogenic, 2 uncertain, and 0 benign)		
**c.281G>C**	p.Gly94Ala	rs121912438	24 missense/in-frame/non-synonymous variants (22 pathogenic, 2 uncertain, and 0 benign)		
**c.284T>C**	p.Val95Ala	rs202198235	22 missense/in-frame/non-synonymous variants (20 pathogenic, 2 uncertain, and 0 benign)		
**c.286G>A**	p.Ala96Thr	rs1568810686	21 missense/in-frame/non-synonymous variants (19 pathogenic, 2 uncertain, and 0 benign)		
**c.287C>G**	p.Ala96Gly	rs1568810690	21 missense/in-frame/non-synonymous variants (19 pathogenic, 2 uncertain, and 0 benign)		
**c.287C>T**	p.Ala96Val	rs1568810690	21 missense/in-frame/non-synonymous variants (19 pathogenic, 2 uncertain, and 0 benign)		
**c.289G>A**	p.Asp97Asn	rs121912459	20 missense/in-frame/non-synonymous variants (18 pathogenic, 2 uncertain, and 0 benign)		
**c.290A>T**	p.Asp97Val	rs1555836803	20 missense/in-frame/non-synonymous variants (18 pathogenic, 2 uncertain, and 0 benign)		

**c.292G>A**	p.Val98Met	rs1555836806	21 missense/in-frame/non-synonymous variants (19 pathogenic, 2 uncertain, and 0 benign)		
**c.292G>C**	p.Val98Leu	rs1555836806	21 missense/in-frame/non-synonymous variants (19 pathogenic, 2 uncertain, and 0 benign)		
**c.298A>G**	p.Ile100Val	rs760740095	18 missense/in-frame/non-synonymous variants (16 pathogenic, 2 uncertain, and 0 benign)		
**c.301G>A**	p.Glu101Lys	rs76731700	20 missense/in-frame/non-synonymous variants (17 pathogenic, 3 uncertain, and 0 benign)		
**c.301G>C**	p.Glu101Gln		20 missense/in-frame/non-synonymous variants (17 pathogenic, 3 uncertain, and 0 benign)		
**c.302A>G**	p.Glu101Gly	rs121912439	20 missense/in-frame/non-synonymous variants (17 pathogenic, 3 uncertain, and 0 benign)		
**c.304G>A**	p.Asp102Asn	rs1568810715	21 missense/in-frame/non-synonymous variants (17 pathogenic, 4 uncertain, and 0 benign)		
**c.304G>C**	p.Asp102His	rs1568810715	21 missense/in-frame/non-synonymous variants (17 pathogenic, 4 uncertain, and 0 benign)		
**c.304G>T**	p.Asp102Ty	rs1568810715	21 missense/in-frame/non-synonymous variants (17 pathogenic, 4 uncertain, and 0 benign)		
**c.305A>G**	p.Asp102Gly	rs1568810721	21 missense/in-frame/non-synonymous variants (17 pathogenic, 4 uncertain, and 0 benign)		
**c.313A>T**	p.Ile105Phe	rs121912445		18 missense/in-frame/non-synonymous variants (13 pathogenic, 5 uncertain, and 0 benign)	
**c.317C>T**	p.Ser106Leu	rs1378590183		18 missense/in-frame/non-synonymous variants (14 pathogenic, 4 uncertain, and 0 benign)	
**c.319C>G**	p.Leu107Val	rs121912440	19 missense/in-frame/non-synonymous variants (15 pathogenic, 4 uncertain, and 0 benign)		
**c.319C>T**	p.Leu107Phe	rs121912440	19 missense/in-frame/non-synonymous variants (15 pathogenic, 4 uncertain, and 0 benign)		

**c.326G>A**	p.Gly109Glu	rs1359299834	21 missense/in-frame/non-synonymous variants (17 pathogenic, 4 uncertain, and 0 benign)		
**c.326G>T**	p.Gly109Val	rs1359299834	21 missense/in-frame/non-synonymous variants (17 pathogenic, 4 uncertain, and 0 benign)		
**c.328G>T**	p.Asp110Tyr	rs567432143	20 missense/in-frame/non-synonymous variants (16 pathogenic, 4 uncertain, and 0 benign)		
**c.335G>A**	p.Cys112Tyr	rs1601158483	19 missense/in-frame/non-synonymous variants (15 pathogenic, 4 uncertain, and 0 benign)		
**c.338T>C**	p.Ile113Thr	rs74315452	19 missense/in-frame/non-synonymous variants (15 pathogenic, 4 uncertain, and 0 benign)		
**c.339C>G**	p.Ile113Met	rs1299542356		18 missense/in-frame/non-synonymous variants (14 pathogenic, 4 uncertain, and 0 benign)	
**c.340A>T**	p.Ile114Phe	rs1568810780		18 missense/in-frame/non-synonymous variants (14 pathogenic, 4 uncertain, and 0 benign)	
**c.341T>C**	p.Ile114Thr	rs121912441		18 missense/in-frame/non-synonymous variants (14 pathogenic, 4 uncertain, and 0 benign)	
**c.344G>C**	p.Gly115Ala	rs1568810789		17 missense/in-frame/non-synonymous variants (13 pathogenic, 4 uncertain, and 0 benign)	
**c.346C>G**	p.Arg116Gly	rs1301635320		15 missense/in-frame/non-synonymous variants (12 pathogenic, 3 uncertain, and 0 benign)	
**c.346C>T**	p.Arg116Cys	rs1301635320		15 missense/in-frame/non-synonymous variants (12 pathogenic, 3 uncertain, and 0 benign)	
**c.350C>G**	p.Thr117Arg	rs1568810800		15 missense/in-frame/non-synonymous variants (12 pathogenic, 3 uncertain, and 0 benign)	
**c.352C>G**	p.Leu118Val	rs199474723		13 missense/in-frame/non-synonymous variants (11 pathogenic, 2 uncertain, and 0 benign)	
**c.355G>C**	p.Val119Leu	rs1235629842		12 missense/in-frame/non-synonymous variants (11 pathogenic, 1 uncertain, and 0 benign)	

**c.355G>T**	p.Val119Leu	rs1235629842		12 missense/in-frame/non-synonymous variants (11 pathogenic, 1 uncertain, and 0 benign)	
**c.355G>A**	p.Val119Met			12 missense/in-frame/non-synonymous variants (11 pathogenic, 1 uncertain, and 0 benign)	
**c.358G>T**	p.Val120Phe			12 missense/in-frame/non-synonymous variants (11 pathogenic, 1 uncertain, and 0 benign)	
**c.358G>C**	p.Val120Leu	rs1457889952		9 missense/in-frame/non-synonymous variants (7 pathogenic, 2 uncertain, and 0 benign)	
**c.361C>A**	p.His121Asn			9 missense/in-frame/non-synonymous variants (7 pathogenic, 2 uncertain, and 0 benign)	
**c.362A>T**	p.His121Leu	rs1410925719		13 missense/in-frame/non-synonymous variants (11 pathogenic, 2 uncertain, and 0 benign)	
**c.374A>C**	p.Asp125Ala	rs1568811366		11 missense/in-frame/non-synonymous variants (8 pathogenic, 3 uncertain, and 0 benign)	
**c.374A>T**	p.Asp125Val	rs1568811366		11 missense/in-frame/non-synonymous variants (8 pathogenic, 3 uncertain, and 0 benign)	
**c.376G>C**	p.Asp126His	rs1568811372		12 missense/in-frame/non-synonymous variants (9 pathogenic, 3 uncertain, and 0 benign)	
**c.376G>A**	p.Asp126Asn			12 missense/in-frame/non-synonymous variants (9 pathogenic, 3 uncertain, and 0 benign)	
**c.377A>C**		rs1164911383		12 missense/in-frame/non-synonymous variants (9 pathogenic, 3 uncertain, and 0 benign)	
**c.380T>C**	p.Leu127Ser	rs121912454		13 missense/in-frame/non-synonymous variants (10 pathogenic, 3 uncertain, and 0 benign)	
**c.382G>C**	p.Gly128Arg	rs1568811389		13 missense/in-frame/non-synonymous variants (10 pathogenic, 3 uncertain, and 0 benign)	
**c.385A>G**	p.Lys129Glu			12 missense/in-frame/non-synonymous variants (9 pathogenic, 3 uncertain, and 0 benign)	

**c.400G>A**	p.Glu134Lys			15 missense/in-frame/non-synonymous variants (11 pathogenic, 4 uncertain, and 0 benign)	
**c.401A>T**	p.Glu134Val	rs1568811426		15 missense/in-frame/non-synonymous variants (11 pathogenic, 4 uncertain, and 0 benign)	
**c.403A>G**	p.Ser135Gly	rs1555836932		12 missense/in-frame/non-synonymous variants (8 pathogenic, 4 uncertain, and 0 benign)	
**c.404G>A**	p.Ser135Asn	rs121912451		12 missense/in-frame/non-synonymous variants (8 pathogenic, 4 uncertain, and 0 benign)	
**c.404G>C**	p.Ser135Thr	rs121912451		12 missense/in-frame/non-synonymous variants (8 pathogenic, 4 uncertain, and 0 benign)	
**c.412A>G**	p.Thr138Ala	rs1568811445		16 missense/in-frame/non-synonymous variants (12 pathogenic, 4 uncertain, and 0 benign)	
**c.413C>G**	p.Thr138Arg	rs1568811454		16 missense/in-frame/non-synonymous variants (12 pathogenic, 4 uncertain, and 0 benign)	
**c.416G>A**	p.Gly139Glu	rs1568811464		17 missense/in-frame/non-synonymous variants (13 pathogenic, 4 uncertain, and 0 benign)	
**c.418A>C**	p.Asn140His	rs1568811471	19 missense/in-frame/non-synonymous variants (15 pathogenic, 4 uncertain, and 0 benign)		
**c.418A>G**	p.Asn140Asp	rs1568811471	19 missense/in-frame/non-synonymous variants (15 pathogenic, 4 uncertain, and 0 benign)		
**c.420C>A**	p.Asn140Lys	rs1804449	20 missense/in-frame/non-synonymous variants (16 pathogenic, 4 uncertain, and 0 benign)		
**c.422C>G**	p.Ala141Gly	rs1555836937	21 missense/in-frame/non-synonymous variants (16 pathogenic, 5 uncertain, and 0 benign)		
**c.424G>A**	p.Gly142Arg		23 missense/in-frame/non-synonymous variants (17 pathogenic, 6 uncertain, and 0 benign)		
**c.425G>A**	p.Gly142Glu	rs1568811489	22 missense/in-frame/non-synonymous variants (17 pathogenic, 5 uncertain, and 0 benign)		

**c.425G>C**	p.Gly142Ala	rs1568811489	22 missense/in-frame/non-synonymous variants (17 pathogenic, 5 uncertain, and 0 benign)		
**c.434T>C**	p.Leu145Ser	rs121912446	23 missense/in-frame/non-synonymous variants (16 pathogenic, 7 uncertain, and 0 benign)		
**c.435G>C**	p.Leu145Phe	rs1482760341	23 missense/in-frame/non-synonymous variants (16 pathogenic, 7 uncertain, and 0 benign)		
**c.436G>A**	p.Ala146Thr	rs121912447	23 missense/in-frame/non-synonymous variants (16 pathogenic, 7 uncertain, and 0 benign)		
**c.437C>A**	p.Ala146Asp	rs1131690781	23 missense/in-frame/non-synonymous variants (16 pathogenic, 7 uncertain, and 0 benign)		
**c.437C>G**	p.Ala146Gly	rs1131690781	23 missense/in-frame/non-synonymous variants (16 pathogenic, 7 uncertain, and 0 benign)		
**c.439T>C**	p.Cys147Arg	rs1568811515	21 missense/in-frame/non-synonymous variants (15 pathogenic, 6 uncertain, and 0 benign)		
**c.442G>C**	p.Gly148Arg	rs1568811520	20 missense/in-frame/non-synonymous variants (15 pathogenic, 5 uncertain, and 0 benign)		
**c.442G>A**	p.Gly148Ser		20 missense/in-frame/non-synonymous variants (15 pathogenic, 5 uncertain, and 0 benign)		
**c.442G>T**	p.Gly148Cys		20 missense/in-frame/non-synonymous variants (15 pathogenic, 5 uncertain, and 0 benign)		
**c.443G>A**	p.Gly148Asp	rs1555836950	20 missense/in-frame/non-synonymous variants (15 pathogenic, 5 uncertain, and 0 benign)		
**c.445G>A**	p.Val149Ile	rs567511139		19 missense/in-frame/non-synonymous variants (14 pathogenic, 5 uncertain, and 0 benign)	
**c.446T>G**	p.Val149Gly	rs1476760624		17 missense/in-frame/non-synonymous variants (12 pathogenic, 5 uncertain, and 0 benign)	
**c.446T>C**	p.Val149Ala			17 missense/in-frame/non-synonymous variants (12 pathogenic, 5 uncertain, and 0 benign)	

**c.448A>G**	p.Ile150Val	rs1169917994		16 missense/in-frame/non-synonymous variants (12 pathogenic, 4 uncertain, and 0 benign),	
**c.449T>C**	p.Ile150Thr	rs1424014997		16 missense/in-frame/non-synonymous variants (12 pathogenic, 4 uncertain, and 0 benign),	
**c.455T>C**	p.Ile152Thr	rs121912449		16 missense/in-frame/non-synonymous variants (12 pathogenic, 4 uncertain, and 0 benign	
**c.455T>G**	p.Ile152Ser			16 missense/in-frame/non-synonymous variants (12 pathogenic, 4 uncertain, and 0 benign	
**c.457G>A**	p.Ala153Thr	rs747094021		16 missense/in-frame/non-synonymous variants (12 pathogenic, 4 uncertain, and 0 benign)	
