# Supplementary material for: SOD-1 Variants in Amyotrophic Lateral Sclerosis: Systematic Re-Evaluation According to ACMG-AMP Guidelines
Source: Genes (Basel). 2022 Mar 18;13(3):537. doi: 10.3390/genes13030537 (PMC8955492; doi:10.3390/genes13030537)
Supplement: Supplementary file 1 [file genes-13-00537-s001.zip › Supplementary Table S3.pdf]

Supplementary Table S3. List of *in vivo* and *in vitro* functional studies considered for the PS3 criterion attribution.

In vivo **functional studies** [transgenic mouse, Zebrafish, D. Melanogaster, rat, C. elegans]				
**Protein change (NP_000445.1)**		**PMID**	**Author's**	**Reference**
p.Gly94Ala	G94A	11717358	Nagai et al., 2001	39
		31771229	Li et al., 2019	40
		33921446	Liguori et al., 2021	41
		34830115	Bonifacino et al., 2021	31
p.His47Arg	H47R	17549011	Sasaki, et al., 2007	42
		34830115	Bonifacino et al., 2021	31
		11717358	Nagai et al., 2001	39
p.Asp91Val	D91A	17146286	Jonnson et al., 2006	43
		33921446	Liguori et al., 2021	41
p.Ala5Val	A5V	33921446	Liguori et al., 2021	41
p.Ala5Thr	A5T	22745481	Hsueh-Ning Liu et al., 2012	44
p.Leu85Val	L85V	33921446	Liguori et al., 2021	41
p.Gly86Arg	G86R	34830115	Bonifacino et al., 2021	31
		30296255	Baskoylu et al., 2018	45
		9052802	Bruijn et al., 1997	46
		27974499	Şahin et al., 2017	47
p.Gly38Arg	G38R	34830115	Bonifacino et al., 2021	31
		27974499	Şahin et al., 2017	47
		19660548	Witan et al., 2009	48
p.Gly94Arg	G94R	34830115	Bonifacino et al., 2021	31
		20504969	Ramesh et al., 2010	49
p.Asp83Gly	D83G	34830115	Bonifacino et al., 2021	31

In vitro functional studies<br>[Fibroblast, NSC34, N2a, EC293FT, iPSC]				
**Protein change (NP_000445.1)**		**PMID**	**Author's**	**Reference**
p.Gly94Ala	G94A	26919046	Keskin et al., 2016	50
		30887850	Lin et al., 2019	51
		19483195	Prudencio et al., 2009	52
p.His47Arg	H47R	26919046	Keskin et al., 2016	50
		18006498	Cozzolino et al., 2008	54
p.Asp91Val	D91A	26919046	Keskin et al., 2016	61
		30550341	Walczak et al., 2019	54
		19483195	Prudencio et al., 2009	52
p.Ala5Val	A5V	26919046	Keskin et al., 2016	50
		30887850	Lin et al., 2019	51
		19483195	Prudencio et al., 2009	52
p.Ala5Thr	A5T	19483195	Prudencio et al., 2009	52
p.Leu85Val	L85V	19483195	Prudencio et al., 2009	52
p.Gly86Arg	G86R	20962037	Wang et al., 2011	55
p.Gly94Arg	G94R	19483195	Prudencio et al., 2009	52
		18552350	Furukawa et al., 2008	58
p.Glu101Lys	E101K	19483195	Prudencio et al., 2009	52
		25600987	Salehi et al., 2015	59
p.Asn140Lys	N140K	19483195	Prudencio et al., 2009	52
p.Cys147Arg	C147R	18006498	Cozzolino et al., 2008	53
p.Val149Gly	V149G	19483195	Prudencio et al., 2009	52
		24253732	McAlary et al., 2013	60

		27867347	McAlary et al., 2016	61
p.Leu39Arg	L39R	32676988	Rajpurohit et al., 2020	62
		28463106	Zhong et al., 2017	63
p.Val15Gly	V15G	19483195	Prudencio et al., 2009	52
p.Ile114Thr	I114T	24439480	Allen et al., 2014	57
p.Gly94Asp	G94D	19483195	Prudencio et al., 2009	52
p.Gly94Ser	G94S	19483195	Prudencio et al., 2009	52
p.Asp125Val	D125V	19483195	Prudencio et al., 2009	52
p.Val15Met	V15M	19483195	Prudencio et al., 2009	52
p.Glu22Gly	E22G	19483195	Prudencio et al., 2009	52
p.Glu22Lys	E22K	19483195	Prudencio et al., 2009	52
p.Gly42Asp	G42D	19483195	Prudencio et al., 2009	52
p.Glu101Gly	E101G	19483195	Prudencio et al., 2009	52
p.Leu145Phe	L145F	19483195	Prudencio et al., 2009	52
p.Gly42Ser	G42S	19483195	Prudencio et al., 2009	52
p.Asp102Gly	D102G	19483195	Prudencio et al., 2009	52
p.Leu145Ser	L145S	19483195	Prudencio et al., 2009	52
p.Asn87Ser	N87S	26919046	Keskin et al., 2016	50
p.Leu118Val	L118V	26919046	Keskin et al., 2016	50
p.Pro75Ser	P75S	30887850	Lin et al., 2019	51
