# Supplementary material for: SOD-1 Variants in Amyotrophic Lateral Sclerosis: Systematic Re-Evaluation According to ACMG-AMP Guidelines
Source: Genes (Basel). 2022 Mar 18;13(3):537. doi: 10.3390/genes13030537 (PMC8955492; doi:10.3390/genes13030537)
Supplement: Supplementary file 1 [file genes-13-00537-s001.zip › Supplementary Table S4.pdf]

**Supplementary Table S4**. Characteristics of the *SOD1* variants relevant to the PVS1 criterion.

| _Coding change (NM_000454.5) | Protein change (NP_000445.1) | Consequence Type | Predict/Not predict to undergo NMD | Location. Protein Position. Splice distance. |
|---|---|---|---|---|
| c.301G>T | p.Glu101Ter | stop gained | Not predict to undergo NMD | Nonsense mutation in exon 4, position 2 of 118. Protein position 101 of 155. Splice distance -57. |
| c.380T>A | p.Leu127Ter | stop gained | Not predict to undergo NMD | Nonsense in exon 5, position 23 of 461. Protein position 127 of 155. Splice distance 23 |
| c.441T>A | p.Cys147Ter | nonsense variant | Not predict to undergo NMD | Nonsense in exon 5, position 84 of 461. Protein position 147 of 155. Splice distance 84. |
| c.424G>T | p.Gly142Ter | stop gained | Not predict to undergo NMD | Nonsense in exon 5, position 67 of 461. Protein position 142 of 155. Splice distance 67. |
| c.383_384insACCC | p.Lys129ProfsTer6 | frameshift variant | Not predict to undergo NMD | Frameshift variants in exon 5 before poistion 27 of 461. Protein position 128 of 155. Splice distance 27. |
| c.384_385insTGGG | p.Lys129TrpfsTer6 | frameshift variant | Not predict to undergo NMD | Frameshift variants in exon 5 before poistion 28 of 461. Protein position 129 of 155. Splice distance 28. |
| c.409A>T | p.Lys137Ter | nonsense variant | Not predict to undergo NMD | Nonsense variant in exon 4, position 52 of 461. Protein position 137 of 155. Splice distance 52. |
| c.275_276delAA | p.Lys92ArgfsTer9 | frameshift variant | Predict to undergo NMD | Frameshift variant in exon 4 position 36-37 of 118. Protein position 92 of 155. Splice distance 36 |
| c.320_321insT | p.Ser108LeufsTer15 | frameshift variant | Not predict to undergo NMD | Frameshift variant in exon 4 before position 82 of 118. Protein position 107 of 155. Splice distance -38 |
| c.383_392dup | p.Asn132GlnfsTer5 | frameshift variant | Not predict to undergo NMD | Frameshift variant in exon 5 position 36 of 461. Protein position 131 of 155. Splice distance 36 |
| c.335dup | p.Cys112TrpfsTer11 | frameshift variant | Not predict to undergo NMD | Frameshit variant in exon 4 before position 97 of 118 Protein position 112 of 155. Splice distance -23, |
