# Supplementary material for: SOD-1 Variants in Amyotrophic Lateral Sclerosis: Systematic Re-Evaluation According to ACMG-AMP Guidelines
Source: Genes (Basel). 2022 Mar 18;13(3):537. doi: 10.3390/genes13030537 (PMC8955492; doi:10.3390/genes13030537)
Supplement: Supplementary file 1 [file genes-13-00537-s001.zip › Supplementary Table S5.pdf]

Supplementary Table S5. List of *SOD1* variants and concordance/discordance between open databases and re-classification.

Coding change (NM_000454.5)	Protein change (NP_000445.1)	Variant ID	LOVD	ClinVar	Re-classification
c.59A>G	p.Asn20Ser	rs768029813	Likely benign	Conflicting interpretations of pathogenicity	Uncertain significance
c.68A>T	p.Gln23Leu	rs1169198442	Likely pathogenic .Pathogenic	Likely pathogenic	Likely pathogenic
c.184G>C	p.Gly62Arg	rs1568810268			Likely pathogenic
c.251A>T	p.Asp84Val				Likely pathogenic
c.14C>T	p.Ala5Val	rs121912442		Pathogenic	Pathogenic
c.13G>A	p.Ala5Thr	rs121912444		Pathogenic	Pathogenic
c.13G>T	p.Ala5Ser	rs121912444		Pathogenic	Pathogenic
c.16G>T	p.Val6Leu	rs1568807314			Likely pathogenic
c.23T>A	p.Val8Glu	rs1568807330			Likely pathogenic
c.19T>A	p.Cys7Ser	rs1312702973			Likely pathogenic
c.20G>A	p.Cys7Tyr	rs121912448		Pathogenic	Likely pathogenic
c.26T>A	p.Leu9Gln	rs1568807342	Likely pathogenic		Pathogenic
c.25C>G	p.Leu9Val	rs1568807333			Pathogenic
c.32G>C	p.Gly11Ala	rs1555836167	Pathogenic		Likely pathogenic
c.31G>C	p.Gly11Arg	rs1568807350			Likely pathogenic
c.34G>T	p.Asp12Tyr	rs762628133			Likely pathogenic
c.203T>C	p.Leu68Pro	rs1568810289			Likely pathogenic
c.217G>A	p.Gly73Ser	rs121912455		Pathogenic	Likely pathogenic
c.255G>C	p.Leu85Phe	rs1315541036		Pathogenic	Pathogenic
c.256G>A	p.Gly86Ser	rs121912436		Pathogenic	Pathogenic
c.272A>C	p.Asp91Ala	rs80265967	Uncertain significance	Conflicting interpretations of pathogenicity	Pathogenic
c.281G>A	p.Gly94Asp	rs121912438		Pathogenic	Pathogenic
c.340A>T	p.Ile114Phe	rs1568810780			Likely pathogenic
c.435G>C	p.Leu145Phe	rs1482760341	Pathogenic	Pathogenic	Pathogenic
c.449T>C	p.Ile150Thr	rs1424014997	Pathogenic		Likely pathogenic
c.255G>T	p.Leu85Phe		Pathogenic		Pathogenic
c.256G>C	p.Gly86Arg	rs121912436		Pathogenic	Pathogenic
c.37G>C	p.Gly13Arg	rs121912456		Pathogenic	Likely pathogenic
c.43G>A	p.Val15Met	rs1568807400		Likely pathogenic	Likely pathogenic
c.44T>C	p.Val15Ala	rs1202989817			Likely pathogenic
c.50G>C	p.Gly17Ala	rs1200906022		Pathogenic/ Likely pathogenic	Likely pathogenic
c.49G>A	p.Gly17Ser	rs121912453		Pathogenic	Likely pathogenic
c.62T>G	p.Phe21Cys	rs1555836169			Likely pathogenic

c.64G>A	p.Glu22Lys	rs121912450		Pathogenic	Likely pathogenic
c.65A>G	p.Glu22Gly	rs1568807435			Likely pathogenic
c.89T>C	p.Val30Ala	rs1568809118			Uncertain Significance
c.112G>A	p.Gly38Arg	rs121912431		Pathogenic	Pathogenic
c.116T>A	p.Leu39Gln	rs1555836520			Likely pathogenic
c.115C>G	p.Leu39Val	rs121912432		Likely pathogenic	Likely pathogenic
c.124G>A	p.Gly42Ser	rs121912433		Pathogenic	Likely pathogenic
c.125G>A	p.Gly42Asp	rs121912434		Pathogenic	Likely pathogenic
c.131A>G	p.His44Arg	rs121912435		Pathogenic	Likely pathogenic
c.137T>C	p.Phe46Ser	rs121912457		Pathogenic	Likely pathogenic
c.140A>G	p.His47Arg	rs121912443	Pathogenic	Pathogenic	Pathogenic
c.142G>T	p.Val48Phe	rs1555836523	Pathogenic		Likely pathogenic
c.143T>C	p.Val48Ala	rs1568809169		Uncertain significance	Likely pathogenic
c.146A>G	p.His49Arg	rs1568809172	Pathogenic	Likely pathogenic	Likely pathogenic
c.147T>G	p.His49Gln	rs1568809175			Likely pathogenic
c.148G>A	p.Glu50Lys	rs1568809178		Uncertain significance	Pathogenic
c.164C>G	p.Thr55Arg	rs986277034			Likely pathogenic
c.179G>A	p.Ser60Asn	rs1413388444			Uncertain Significant
c.197A>G	p.Asn66Ser	rs1568810275			Likely pathogenic
c.199C>G	p.Pro67Ala	rs1356474292			Likely pathogenic
c.229G>T	p.Asp77Tyr	rs1601157750		Likely pathogenic	Likely pathogenic
c.230A>T	p.Asp77Val	rs1568810316		Likely pathogenic	Likely pathogenic
c.263T>C	p.Val88Ala	rs1339283341		Likely pathogenic	Pathogenic
c.262G>A	p.Val88Met	rs1568810641		Likely pathogenic	Pathogenic
c.260A>G	p.Asn87Ser	rs11556620		Pathogenic/<br>Likely pathogenic	Pathogenic
c.260A>T	p.Asn87Ile	rs11556620			Likely pathogenic
c.259A>G	p.Asn87Asp	rs1555836792			Likely pathogenic
c.261T>A	p.Asn87Lys	rs1555836793			Likely pathogenic
c.268G>A	p.Ala90Thr	rs1568810660		Likely pathogenic	Pathogenic
c.269C>T	p.Ala90Val	rs1280042397	Likely pathogenic		Pathogenic
c.281G>C	p.Gly94Ala	rs121912438		Pathogenic	Pathogenic
c.280G>A	p.Gly94Ser	rs121912437			Pathogenic
c.280G>T	p.Gly94Cys	rs121912437		Likely pathogenic	Pathogenic
c.280G>C	p.Gly94Arg	rs121912437		Pathogenic	Pathogenic
c.286G>A	p.Ala96Thr	rs1568810686			Likely pathogenic
c.287C>G	p.Ala96Gly	rs1568810690			Likely pathogenic
c.289G>A	p.Asp97Asn	rs121912459		Uncertain significance	Likely pathogenic
c.290A>T	p.Asp97Val	rs1555836803		Likely pathogenic	Likely pathogenic
c.292G>A	p.Val98Met	rs1555836806			Likely pathogenic
c.292G>C	p.Val98Leu	rs1555836806			Likely pathogenic

c.298A>G	p.Ile100Val	rs760740095	Uncertain significance		Uncertain significance
c.302A>G	p.Glu101Gly	rs121912439		Likely pathogenic	Pathogenic
c.242A>G	p.His81Arg	rs121912458		Pathogenic	Likely pathogenic
c.253T>G	p.Leu85Val	rs121912452		Pathogenic	Pathogenic
c.301G>A	p.Glu101Lys	rs76731700		Pathogenic/ Likely pathogenic	Pathogenic
c.301G>T	p.Glu101Ter	rs76731700		Pathogenic	Likely pathogenic
c.313A>T	p.Ile105Phe	rs121912445		Pathogenic	Likely pathogenic
c.305A>G	p.Asp102Gly	rs1568810721			Pathogenic
c.304G>A	p.Asp102Asn	rs1568810715		Likely pathogenic	Pathogenic
c.304G>C	p.Asp102His	rs1568810715		Likely pathogenic	Pathogenic
c.317C>T	p.Ser106Leu	rs1378590183		Pathogenic	Likely pathogenic
c.319C>G	p.Leu107Val	rs121912440		Pathogenic	Likely pathogenic
c.319C>T	p.Leu107Phe	rs121912440		Uncertain significance	Likely pathogenic
c.326G>A	p.Gly109Glu	rs1359299834			Likely pathogenic
c.338T>C	p.Ile113Thr	rs74315452		Likely pathogenic	Pathogenic
c.339C>G	p.Ile113Met	rs1299542356			Likely pathogenic
c.344G>C	p.Gly115Ala	rs1568810789			Likely pathogenic
c.346C>G	p.Arg116Gly	rs1301635320		Pathogenic	Likely pathogenic
c.352C>G	p.Leu118Val	rs199474723		Conflicting interpretations of pathogenicity. Likely pathogenic;Uncertain significance	Likely Pathogenic
c.355G>C	p.Val119Leu	rs1235629842		Uncertain significance	Pathogenic
c.374A>C	p.Asp125Ala	rs1568811366		Uncertain significance	Likely pathogenic
c.374A>T	p.Asp125Val	rs1568811366			Likely pathogenic
c.376G>C	p.Asp126His	rs1568811372		Likely pathogenic	Likely pathogenic
c.380T>A	p.Leu127Ter	rs121912454		Pathogenic	Pathogenic
c.382G>C	p.Gly128Arg	rs1568811389			Likely pathogenic
c.401A>T	p.Glu134Val	rs1568811426			Likely pathogenic
c.404G>A	p.Ser135Asn	rs121912451		Pathogenic	Likely pathogenic
c.413C>G	p.Thr138Arg	rs1568811454			Likely pathogenic
c.416G>A	p.Gly139Glu	rs1568811464		Uncertain significance	Likely pathogenic
c.418A>C	p.Asn140His	rs1568811471			Likely pathogenic
c.420C>A	p.Asn140Lys	rs1804449		Pathogenic	Pathogenic
c.422C>G	p.Ala141Gly	rs1555836937		Uncertain significance	Likely pathogenic
c.425G>A	p.Gly142Glu	rs1568811489			Likely pathogenic
c.434T>C	p.Leu145Ser	rs121912446		Likely pathogenic	Pathogenic
c.436G>A	p.Ala146Thr	rs121912447		Likely pathogenic	Pathogenic

c.437C>A	p.Ala146Asp	rs1131690781		Likely pathogenic	Pathogenic
c.439T>C	p.Cys147Arg	rs1568811515			Pathogenic
c.443G>A	p.Gly148Asp	rs1555836950		Likely pathogenic	Pathogenic
c.442G>C	p.Gly148Arg	rs1568811520		Likely pathogenic	Pathogenic
c.445G>A	p.Val149Ile	rs567511139			Likely pathogenic
c.446T>G	p.Val149Gly	rs1476760624		Likely pathogenic	Likely pathogenic
c.455T>C	p.Ile152Thr	rs121912449		Pathogenic	Likely pathogenic
c.455T>G	p.Ile152Ser			Uncertain significance	Likely pathogenic
c.437C>G	p.Ala146Gly	rs1131690781		Likely pathogenic	Likely pathogenic
c.13_14delGCinsTT	p.Ala5Phe				Likely pathogenic
c.287C>T	p.Ala96Val	rs1568810690			Likely pathogenic
c.335G>A	p.Cys112Tyr	rs1601158483			Likely pathogenic
c.441T>A	p.Cys147Ter				Pathogenic
c.172T>C	p.Cys58Arg	rs1568810255			Uncertain Significant
c.19T>G	p.Cys7Gly	rs1312702973			Likely pathogenic
c.21C>G	p.Cys7Trp		Pathogenic		Likely pathogenic
c.304G>T	p.Asp102Ty	rs1568810715			Likely pathogenic
c.328G>T	p.Asp110Tyr	rs567432143		Uncertain significance	Uncertain significance
c.35A>C	p.Asp12Ala	rs1568807374			Uncertain significance
c.251A>G	p.Asp84Gly	rs1568810615			Likely pathogenic
c.250G>A	p.Asp84Asn	rs1555836789			Likely pathogenic
c.272A>T	p.Asp91Val	rs80265967		Conflicting interpretations of pathogenicity	Pathogenic
c.271G>A	p.Asp91Asn	rs1343616996			Likely pathogenic
c.272_274dupACA	p.Asp91_Lys92insAsn				Likely pathogenic
c.400G>A	p.Glu134Lys				Likely pathogenic
c.122A>G	p.Glu41Gly	rs1568809149		Conflicting interpretations of pathogenicity.Likely pathogenic;Uncertain significance	Likely pathogenic
c.63C>G	p.Phe21Leu	rs1555836170		Uncertain significance	Likely pathogenic
c.137T>G	p.Phe46Cys	rs121912457		Pathogenic	Likely pathogenic
c.193T>C	p.Phe65Leu	rs1030039318			Likely pathogenic
c.326G>T	p.Gly109Val	rs1359299834			Pathogenic
c.425G>C	p.Gly142Ala	rs1568811489			Likely pathogenic
c.424G>T	p.Gly142Ter	rs1319062081			Pathogenic
c.424G>A	p.Gly142Arg				Likely pathogenic
c.442G>A	p.Gly148Ser				Likely pathogenic
c.442G>T	p.Gly148Cys				Likely pathogenic

c.113G>T	p.Gly38Val	rs1555836517			Likely pathogenic
c.217G>T	p.Gly73Cys	rs121912455		Pathogenic	Likely pathogenic
c.218G>A	p.Gly73Asp				Likely pathogenic
c.362A>T	p.His121Leu	rs1410925719			Likely pathogenic
c.139C>G	p.His47Asp	rs748897491			Likely pathogenic
c.341T>C	p.Ile114Thr	rs121912441		Pathogenic	Likely pathogenic
c.448A>G	p.Ile150Val	rs1169917994			Likely pathogenic
c.56_58delTCA	p.Ile19del				Likely pathogenic
c.383_384insACCC	p.Lys129ProfsTer6				Pathogenic
c.384_385insTGGG	p.Lys129TrpfsTer6				Pathogenic
c.409A>T	p.Lys137Ter	rs1555836934			Pathogenic
c.275_276delAA	p.Lys92ArgfsTer9				Pathogenic
c.380T>C	p.Leu127Ser	rs121912454		Pathogenic	Likely pathogenic
c.116T>G	p.Leu39Arg	rs1555836520			Likely pathogenic
c.203T>G	p.Leu68Arg	rs1568810289			Pathogenic
c.418A>G	p.Asn140Asp	rs1568811471			Likely pathogenic
c.200C>G	p.Pro67Arg	rs1568810284			Likely pathogenic
c.199C>T	p.Pro67Ser	rs1356474292			Likely pathogenic
c.69G>C	p.Gln23His	rs1424217272			Likely pathogenic
c.68A>G	p.Gln23Arg	rs1169198442		Likely pathogenic	Likely pathogenic
c.346C>T	p.Arg116Cys	rs1301635320	Likely pathogenic	Pathogenic	Likely pathogenic
c.320_321insT	p.Ser108LeufsTer15			Pathogenic	Pathogenic
c.404G>C	p.Ser135Thr	rs121912451	Pathogenic	Pathogenic	Likely pathogenic
c.179G>T	p.Ser60Ile	rs1413388444			Uncertain Significant
c.240-7T>G		rs1568810602			Uncertain significance
c.358-11A>G		rs369600566		Pathogenic	Uncertain significance
c.358-304C>G		rs1555836889			Uncertain significance
c.350C>G	p.Thr117Arg	rs1568810800			Likely pathogenic
c.412A>G	p.Thr138Ala	rs1568811445		Conflicting interpretations of pathogenicity. Likely pathogenic;Uncertain significance	Likely pathogenic
c.355G>T	p.Val119Leu	rs1235629842		Uncertain significance	Likely pathogenic
c.355G>A	p.Val119Met				Likely pathogenic
c.358G>T	p.Val120Phe				Likely pathogenic
c.446T>C	p.Val149Ala				Likely pathogenic
c.44T>G	p.Val15Gly	rs1202989817			Likely pathogenic
c.95T>C	p.Val32Ala	rs1428716759		Conflicting interpretations of pathogenicity. Likely	Likely pathogenic

				pathogenic. Uncertain significance	
c.284T>C	p.Val95Ala	rs202198235			Likely pathogenic
c.95T>G	p.Val32Gly	rs1428716759			Likely pathogenic
c.112G>C	p.Gly38Arg	rs121912431		Pathogenic	Pathogenic
c.116T>C	p.Leu39Pro				Likely pathogenic
c.66G>C	p.Glu22Asp				Likely pathogenic
c.172T>G	p.Cys58Gly				Uncertain Significant
c.199G>C	p.Pro67Ala	rs1356474292			Likely pathogenic
c.215A>C	p.His72Pro				Likely pathogenic
c.223C>T	p.Pro75Ser				Likely pathogenic
c.224C>G	p.Pro75Arg				Likely pathogenic
c.230A>G	p.Asp77Gly				Likely pathogenic
c.358G>C	p.Val120Leu	rs1457889952			Likely pathogenic
c.301G>C	p.Glu101Gln				Likely pathogenic
c.403A>G	p.Ser135Gly	rs1555836932			Likely pathogenic
c.361C>A	p.His121Asn				Likely pathogenic
c.385A>G	p.Lys129Glu				Likely pathogenic
c.457G>A	p.Ala153Thr	rs747094021		Uncertain significance	Likely pathogenic
c.123A>T	p.Glu41Asp				Likely pathogenic
c.383_392dup	p.Asn132GlnfsTer5		Likely pathogenic. Pathogenic		Pathogenic
c.335dup	p.Cys112TrpfsTer11	rs1568810771		Likely pathogenic	Pathogenic
c.358-10T>G		rs1197141604		Uncertain significance	Uncertain significance
c.377A>C		rs1164911383		Likely pathogenic	Likely pathogenic
c.241C>T	p.His81Tyr				Likely pathogenic
c.376G>A	p.Asp126Asn				Likely pathogenic
