# Supplementary figures and images for: SOD-1 Variants in Amyotrophic Lateral Sclerosis: Systematic Re-Evaluation According to ACMG-AMP Guidelines
Source: Genes (Basel). 2022 Mar 18;13(3):537. doi: 10.3390/genes13030537 (PMC8955492; doi:10.3390/genes13030537)

**Supplementary Figure S2.** Decision tree for functional evidence PS3.

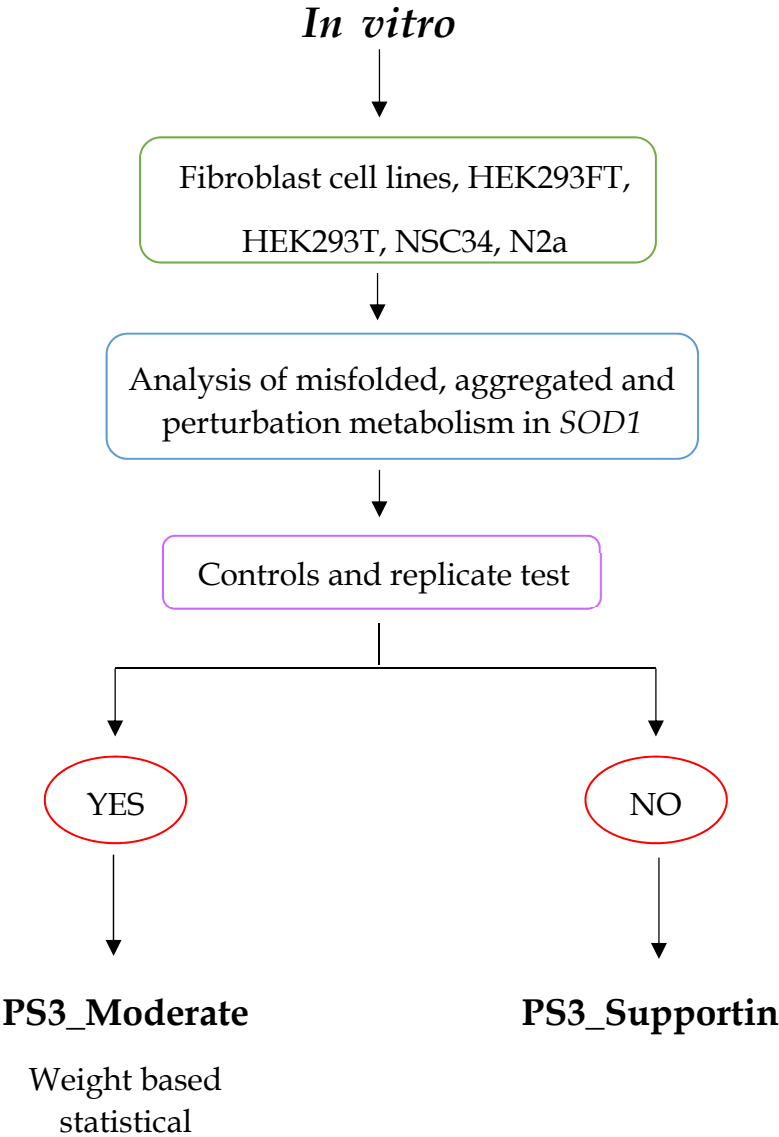

Supplement: Supplementary file 1 [file genes-13-00537-s001.zip › Supplementary Figure S2.pdf]
